# Non-retroviral Endogenous Viral Element Limits Cognate Virus Replication in *Aedes aegypti* Ovaries

**DOI:** 10.1016/j.cub.2020.06.057

**Published:** 2020-09-21

**Authors:** Yasutsugu Suzuki, Artem Baidaliuk, Pascal Miesen, Lionel Frangeul, Anna B. Crist, Sarah H. Merkling, Albin Fontaine, Sebastian Lequime, Isabelle Moltini-Conclois, Hervé Blanc, Ronald P. van Rij, Louis Lambrechts, Maria-Carla Saleh

**Affiliations:** 1 Viruses and RNA Interference Unit, Institut Pasteur, UMR3569, CNRS, Paris, France; 2 Insect-Virus Interactions Unit, Institut Pasteur, UMR2000, CNRS, Paris, France; 3 Collège Doctoral, Sorbonne Université, 75005 Paris, France; 4 Department of Medical Microbiology, Radboud Institute for Molecular Life Sciences, Radboud University Medical Center, Nijmegen, the Netherlands; 5 Laboratory of Clinical and Epidemiological Virology, Rega Institute, Department of Microbiology and Immunology, KU Leuven, Leuven, Belgium

**Keywords:** endogenous viral element, insect-specific flavivirus, cell-fusing agent virus, mosquito, *Aedes aegypti*, RNA interference, siRNA pathway, piRNA pathway, insect immunity, CRISPR-Cas9

## Abstract

Endogenous viral elements (EVEs) are viral sequences integrated in host genomes. A large number of non-retroviral EVEs was recently detected in *Aedes* mosquito genomes, leading to the hypothesis that mosquito EVEs may control exogenous infections by closely related viruses. Here, we experimentally investigated the role of an EVE naturally found in *Aedes aegypti* populations and derived from the widespread insect-specific virus, cell-fusing agent virus (CFAV). Using CRISPR-Cas9 genome editing, we created an *Ae. aegypti* line lacking the CFAV EVE. Absence of the EVE resulted in increased CFAV replication in ovaries, possibly modulating vertical transmission of the virus. Viral replication was controlled by targeting of viral RNA by EVE-derived P-element-induced wimpy testis-interacting RNAs (piRNAs). Our results provide evidence that antiviral piRNAs are produced in the presence of a naturally occurring EVE and its cognate virus, demonstrating a functional link between non-retroviral EVEs and antiviral immunity in a natural insect-virus interaction.

## Highlights

• *Aedes aegypti* harbors EVEs with high sequence identity to a contemporary RNA virus
• EVE-derived piRNAs target genomic viral RNA in infected mosquitoes
• Ablation of EVE results in increased viral replication in *Aedes aegypti* ovaries
• piRNA pathway fulfills antiviral function in presence of EVE and cognate virus

## Graphical Abstract

**Figure:**
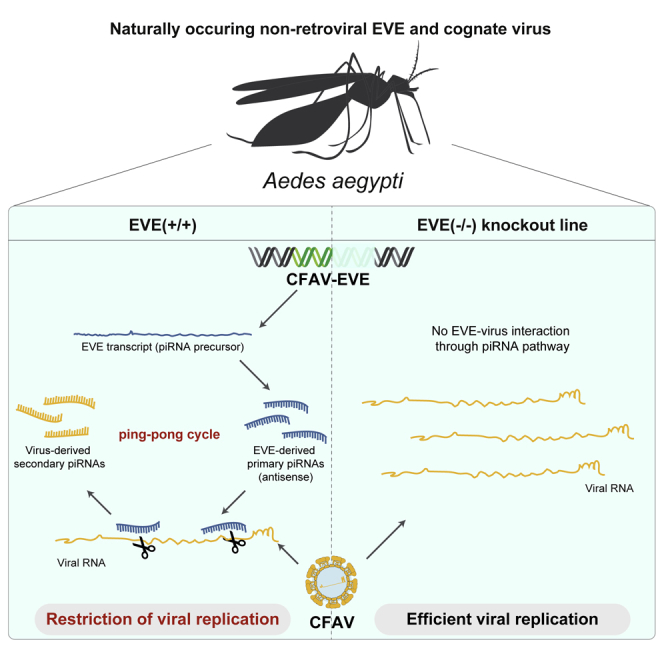

## Teaser

Suzuki, Baidaliuk et al. identify a new endogenous viral element (EVE) in the genome of *Aedes aegypti* mosquitoes that was derived from a currently circulating, insect-specific flavivirus. Using this naturally occurring interaction, they demonstrate that a non-retroviral EVE confers antiviral immunity against its cognate virus via the piRNA pathway.

## Introduction

Host genomes often harbor fragments of viral genomes, referred to as endogenous viral elements (EVEs), that are inherited as host alleles [1]. The best-studied EVEs are derived from mammalian retroviruses, which actively integrate their viral DNA into the host genome during their replication cycle. Retroviral EVEs play important roles in host physiology and antiviral immunity [2]. Recent bioinformatic surveys also identified non-retroviral EVEs in a wide range of animal genomes, albeit their function was only studied in cell lines or protozoa [3, 4, 5, 6, 7, 8, 9, 10, 11]. The endogenization of non-retroviral sequences is presumably mediated by the activity of transposable elements (TEs), which are mobile DNA sequences ubiquitously found in eukaryotic genomes. Non-retroviral EVEs are often integrated in genomic regions surrounded by TEs, suggesting that TEs are involved in the integration and/or expansion of the EVEs [3, 6, 12, 13, 14]. The reverse transcription activity of retrotransposons is the likely mechanism generating non-retroviral DNA from RNA viruses, which are the hypothetical precursors of non-retroviral EVEs [15].

The recent discovery of non-retroviral EVEs in the genomes of mosquito vectors [6, 13, 16] has stimulated studies to elucidate their potential function. In particular, the genomes of the main arthropod-borne virus (arbovirus) vectors *Aedes aegypti* and *Aedes albopictus* harbor hundreds of non-retroviral EVEs predominantly derived from insect-specific viruses of the *Flaviviridae* and *Rhabdoviridae* families [6, 13]. Interest in mosquito EVEs stems from the hypothesis that they may serve as the source of immunological memory against exogenous viruses in insects, as was recently reviewed in [17]. This hypothesis largely relies on the observation that EVEs and their flanking genomic regions serve as templates for P-element-induced wimpy testis (PIWI)-interacting RNAs (piRNAs) [5, 6, 12, 13]. piRNAs are a major class of small RNAs (sRNAs) and are typically generated from genomic loci called piRNA clusters [18]. The piRNA pathway is considered a widely conserved TE-silencing system to prevent deleterious effects of transposition events in eukaryotic genomes, particularly in gonads [19]. In fact, production of EVE-derived piRNAs is observed across a wide range of animals, such as mammals, arthropods, and sea snails [5, 6, 10, 12, 13, 20, 21], in which EVEs are often enriched in piRNA clusters [5, 20, 22]. The predominantly antisense orientation of EVE-derived piRNAs supports the idea that piRNAs could also mediate antiviral immunity by targeting exogenous viral RNA with high levels of sequence identity [6, 13, 22].

The biogenesis of piRNAs and their function as a TE-silencing mechanism to protect genome integrity are well described in the model insect *Drosophila* [23]. piRNAs are characterized by their size of 26–30 nt and distinctive sequence biases. Primary piRNAs typically display a uridine at the first nucleotide position, referred to as 1U bias. Secondary piRNAs overlap primary piRNAs over 10 nt at their 5′ extremity and display an adenine at their 10^th^ nt position, referred to as 10A bias [24, 25]. These characteristics are a consequence of piRNA reciprocal amplification during the ping-pong cycle: (1) primary piRNAs are generated from single-stranded precursor RNA; (2) primary piRNAs guide the cleavage of complementary RNA sequences; (3) secondary piRNAs are generated from the 3′ cleavage products; and (4) secondary piRNAs induce cleavage of piRNA precursor transcripts, which are processed into primary piRNAs. Unlike *Drosophila*, it has been shown that mosquitoes produce virus-derived primary and secondary piRNAs during viral infections [26, 27, 28]. Although most of these observations have been obtained using the *Ae. aegypti* cell line Aag2 and arboviruses, such as dengue or Sindbis viruses, recent studies have shown that viral piRNAs were also found in mosquito cell lines persistently infected with insect-specific viruses, which are not infectious to vertebrates [29, 30].

Whether EVEs can protect insects, and most importantly their germline, from viral infection through the piRNA pathway has not been demonstrated *in vivo*. In mosquitoes, the antiviral activity of viral piRNAs is still debated, and a direct link between EVEs and antiviral activity has yet to be established [31]. One observation casting doubt on this hypothesis is that most arthropod EVEs identified so far are unlikely to serve as sources of antiviral piRNAs because they are not similar enough to currently circulating viruses [5, 20, 22]. Here, we identified a new EVE in *Ae. aegypti* sharing ∼96% nucleotide identity with a wild-type strain of cell-fusing agent virus (CFAV) that we previously isolated from *Ae. aegypti* in Thailand [32]. CFAV is a widespread insect-specific virus infecting *Ae. aegypti* populations around the world [33]. We used this naturally occurring CFAV EVE and the cognate CFAV strain to experimentally investigate the antiviral function of mosquito EVEs in a natural insect-virus interaction. Analysis of sRNAs showed that the CFAV EVE produced primary piRNAs in the absence of CFAV infection. When mosquitoes were infected with CFAV, abundant CFAV-derived piRNAs were produced from the viral genomic regions overlapping with the CFAV EVE. piRNAs displayed a ping-pong signature as well as nucleotide biases consistent with production of EVE-derived primary piRNAs and virus-derived secondary piRNAs. Excision of the CFAV EVE by CRISPR-Cas9 genome engineering resulted in increased CFAV replication in ovaries. Our results provide empirical evidence that a non-retroviral EVE in *Ae. aegypti* contributes to the control of *in vivo* replication of a closely related exogenous virus via the piRNA pathway.

## Results

### Survey of CFAV-Derived EVEs in *Aedes aegypti* Genome Sequences

**Figure 1:**
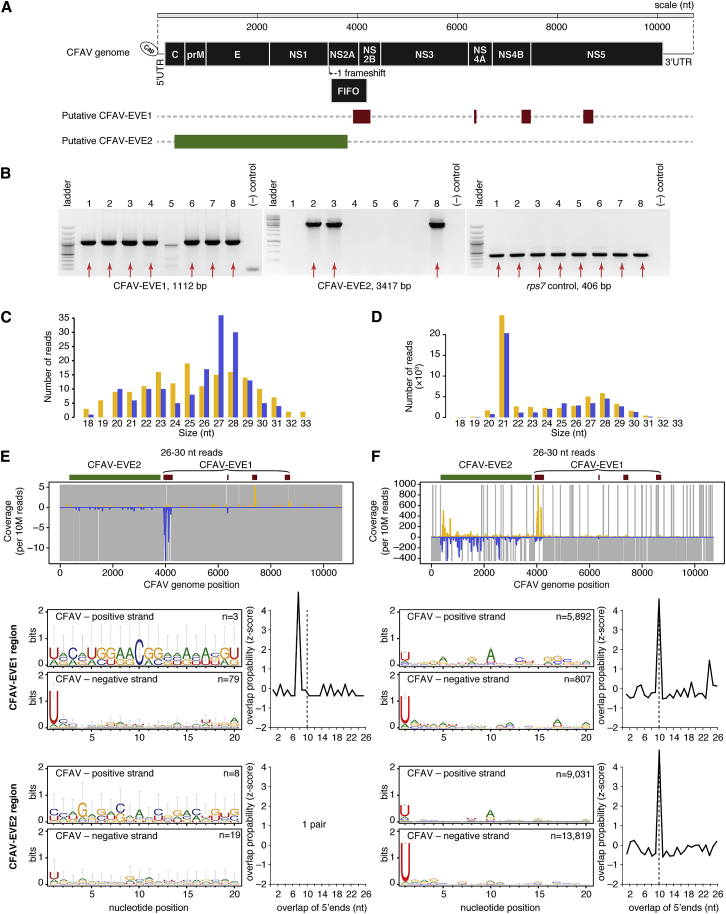
CFAV-Derived Endogenous Viral Elements Interact with Natural CFAV Infection through the piRNA Pathway (A) The schematic represents two potential CFAV EVE structures detected in publicly available *Ae. aegypti* sequences. (B) The presence of putative CFAV-EVE1 and CFAV-EVE2 in eight mosquitoes from the same outbred colony was verified by PCR with primers specific to CFAV-EVE1 (left), CFAV-EVE2 (middle), and *rps7* gene control (right). (C and D) Size distribution of sRNAs mapping to the CFAV genome from naturally CFAV-uninfected (C) and CFAV-infected (D) mosquitoes from the outbred colony. (E and F) Analysis of CFAV-derived piRNAs from naturally CFAV-uninfected (E) and CFAV-infected (F) mosquitoes from the outbred colony. Mapping of 26- to 30-nt sRNAs (top), sequence logos of 26- to 30-nt sRNAs (bottom left), and overlap probability of 26- to 30-nt sRNAs (bottom right) is shown. Sequence logos and overlap probability for CFAV-EVE1 were restricted to the NS2 region. In (C)–(F), positive- and negative-sense reads with respect to the reference CFAV genome are shown in yellow and blue, respectively. Uncovered nucleotides are represented by gray lines. See also Data S1, Figure S1, Table S1, Table S2, and Table S5.

In order to inventory CFAV-derived EVEs, we used BLAST search to identify CFAV-like sequences in publicly available *Ae. aegypti* genome assemblies, RNA sequencing data, and whole-genome sequencing data. We identified several potential EVE structures based on samples for which reads aligned only to segments of the CFAV genome, in addition to samples for which reads covered the entire CFAV genome, presumably representing true CFAV infections (Data S1). The predicted structure of two of these putative EVEs, which we designated CFAV-EVE1 and CFAV-EVE2, was obtained by *de novo* assembly (Figure 1A). These two putative EVEs were confirmed in an outbred *Ae. aegypti* colony derived from a wild population in Thailand and maintained in our laboratory since 2013. Using specific primer sets (Table S1), we detected CFAV-EVE1 and CFAV-EVE2 in 7 out of 8 and in 3 out of 8 individuals, respectively, in this outbred mosquito colony (Figure 1B).

### CFAV-EVEs Produce piRNAs that Interact with Viral RNA from a Natural CFAV Infection

Our outbred *Ae. aegypti* colony from Thailand is naturally infected with a wild-type strain of CFAV, which we previously isolated and named CFAV-KPP [32]. Only a fraction of the mosquitoes in this colony are naturally infected, allowing us to investigate whether the CFAV EVEs produce piRNAs in the presence or absence of a natural CFAV infection. We sequenced sRNA libraries from both naturally infected and uninfected mosquito pools to examine sRNA production and, specifically, EVE-derived and virus-derived piRNA production. In uninfected mosquitoes, the size distribution of the sRNA reads mapping to the CFAV-KPP genome sequence (Figure 1C) showed production of sRNAs of 26–30 nt in size with 1U bias, indicating that they are primary piRNAs generated mainly from the CFAV-EVE1 NS2 fragment and, to a lesser extent, from the CFAV-EVE2 (Figure 1E). The lack of virus-derived 21-nt small interfering RNAs (siRNAs) confirmed the lack of CFAV infection in these mosquitoes (Figures 1C and S1A). In contrast, the sRNA size profile of mosquitoes naturally infected with CFAV-KPP showed abundant production of virus-derived siRNAs (Figures 1D and S1B). The CFAV-infected mosquitoes also harbored positive-stranded (+) CFAV-derived piRNAs, in addition to more abundant negative-stranded (−) primary piRNAs derived from both EVEs (Figure 1F) relative to the uninfected mosquitoes (Figure 1E). The presence of the 10A bias in (+) piRNAs and the 10-nt overlap probability between piRNA reads mapping to opposite strands was consistent with production of secondary virus-derived (+) piRNAs potentially triggered by EVE-derived (−) piRNAs, likely resulting in ping-pong amplification (Figure 1F). Thus, sRNA profiles in our outbred *Ae. aegypti* colony showed that the RNA transcribed from CFAV EVEs interacts with the viral RNA of a natural CFAV infection via the piRNA pathway.

### piRNAs from CFAV-EVE1 Interact with Viral RNA during CFAV Experimental Infection

**Figure 2:**
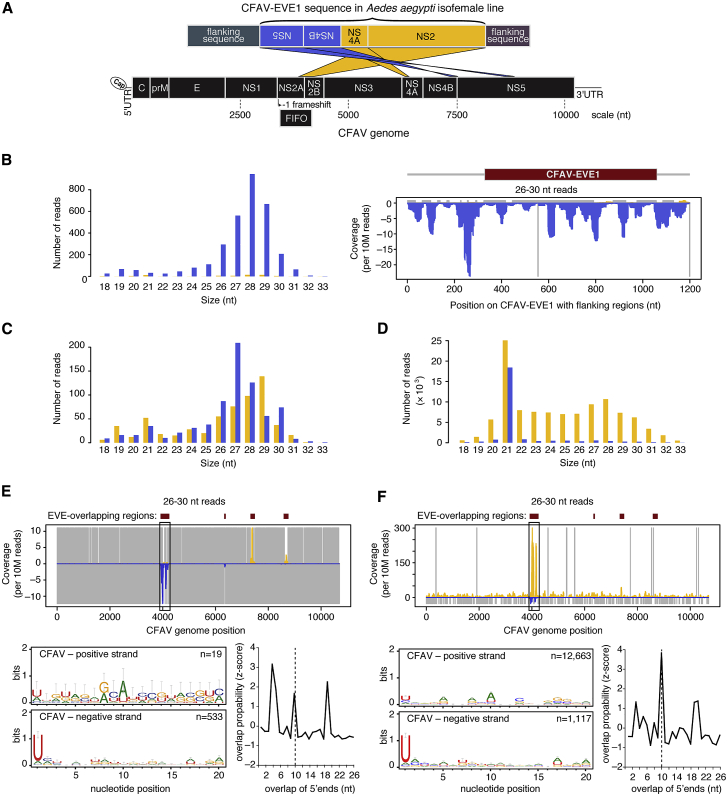
CFAV-EVE1 Interacts with Experimental CFAV Infection through the piRNA Pathway (A) Schematic of the CFAV-EVE1 structure in the CFAV-free isofemale line represented as the alignment of the EVE locus in the *Ae. aegypti* genome assembly AaegL3 (top) to the genome of the CFAV-KPP isolate (bottom). CFAV-EVE1 comprises four different regions of the CFAV genome. Yellow and blue colors indicate forward and reverse strands, respectively, according to the transcription direction in the supercontig. (B) Production of piRNAs from CFAV-EVE1 in the CFAV-free isofemale line, represented as the size distribution (left) and alignment to the CFAV-EVE-1 locus (right). Blue color corresponds to negative-sense reads with respect to the mapping reference. (C and D) Size distribution of sRNAs mapping to the CFAV genome from experimentally CFAV-uninfected (C) and CFAV-infected (D) mosquitoes from the isofemale line. (E and F) Analysis of CFAV-derived piRNAs from experimentally CFAV-uninfected (E) and CFAV-infected (F) mosquitoes from the isofemale line. Mapping of 26- to 30-nt sRNAs (top), sequence logos of 26- to 30-nt sRNAs (bottom left), and overlap probability of 26- to 30-nt sRNAs (bottom right) is shown. Sequence logos and overlap probability were restricted to the NS2 region. In (C)–(F), positive- and negative-sense reads with respect to the reference CFAV genome are shown in yellow and blue, respectively. Uncovered nucleotides are represented by gray lines. See also Figure S2 and Table S5.

To experimentally demonstrate the role of EVEs in antiviral immunity, we took advantage of a CFAV-free isofemale line of *Ae. aegypti* from Thailand maintained in our laboratory since 2010 [34, 35]. We sequenced the whole genome of this isofemale line and only detected the presence of CFAV-EVE1 in the absence of other CFAV EVEs. CFAV-EVE1 was fully reconstructed from the newly obtained genomic data (Figure 2A; Table S2). The structure of CFAV-EVE1 in the isofemale line was consistent with the structure predicted from our bioinformatic survey (Figure 1A). CFAV-EVE1 consists of four adjacent fragments that correspond to the following CFAV genomic regions: NS5; NS4B-NS5; NS4A; and NS2A-NS2B/FIFO (designated as NS2 hereafter for simplicity). The CFAV-EVE1 sequence contains multiple start and stop codons in all six open reading frames. Moreover, two fragments (NS2 and NS4A) are inserted in opposite direction relative to the other EVE fragments, making it unlikely that functional viral peptides are effectively translated. We tested 31 individual mosquitoes from the isofemale line and found that 28 (90%; 95% confidence interval 73%–97%) were positive for CFAV-EVE1. As previously reported for other EVEs [5, 6, 12, 13], CFAV-EVE1 and its flanking regions produced abundant antisense piRNAs (Figure 2B) when aligned to the isofemale line genome sequence. This observation indicates that CFAV-EVE1 is likely transcribed as a part of a longer piRNA precursor.

The CFAV-EVE1 sequence of the isofemale line shared ∼96% nucleotide identity with the CFAV-KPP genome, ranging from 94.6% to 98.8% among the different CFAV-EVE1 fragments (Table S2). To experimentally confirm our observations from naturally infected mosquitoes (Figure 1), we investigated the interaction between CFAV-EVE1 and CFAV-KPP in the isofemale line (Figures 2C and 2D). In the absence of CFAV infection and as a consequence of the dual orientation of the CFAV-EVE1 fragments, EVE-derived piRNAs from the NS2 and NS4A regions were in antisense orientation, whereas EVE-derived piRNAs from the NS4B and NS5 regions were in sense orientation relative to the genome sequence of CFAV. We observed the most pronounced production of 1U biased, antisense primary piRNAs in the NS2 region (black frame in top panel of Figure 2E). When mosquitoes were intrathoracically inoculated with CFAV-KPP stock, the sRNA size profile (Figure 2D) showed abundant production of virus-derived siRNAs (21 nt) and also (+) CFAV-derived piRNAs corresponding to the CFAV-EVE1 genomic region of CFAV, in addition to (−) primary piRNAs derived from the EVE. As the NS2 region is the most abundantly covered by both sense and antisense piRNAs, we used this region (black frame in top panel of Figure 2F) to check for 10A bias as well as ping-pong signature. The 10-nt overlap of 5′ ends was consistent with active ping-pong amplification of the piRNAs in the NS2 region. In addition, analysis of the reads that unambiguously mapped to either the CFAV-KPP genome or to the CFAV-EVE1 sequence revealed that the vast majority of the piRNA reads derived from the CFAV-KPP genome were (+) piRNAs (Figure S2A), whereas almost all of the (−) piRNA reads derived from the CFAV-EVE1 (Figure S2B). It is worth noting that, despite a similar abundance of EVE-derived primary piRNAs from the NS2 and NS4B regions in the absence of infection (Figure 2E), there is no evidence for amplification of piRNAs from the NS4B region during infection (Figure 2F). This suggests that the CFAV (−) RNA is not accessible or abundant enough for PIWI proteins loaded with primary piRNAs to initiate the ping-pong cycle.

Altogether, these results confirmed that CFAV-EVE1 produces piRNAs that target viral RNA and engage in a ping-pong cycle during experimental CFAV infection. The ability to experimentally infect the mosquito isofemale line carrying only CFAV-EVE1 with CFAV-KPP allowed us to directly address the role of non-retroviral EVEs in antiviral immunity. This system recapitulated, under laboratory conditions, a unique situation found in nature (i.e., mosquitoes carrying an EVE that are infected or uninfected with a cognate virus).

### Genome Engineering of a CFAV-EVE1 Knockout Line of *Aedes aegypti*

**Figure 3:**
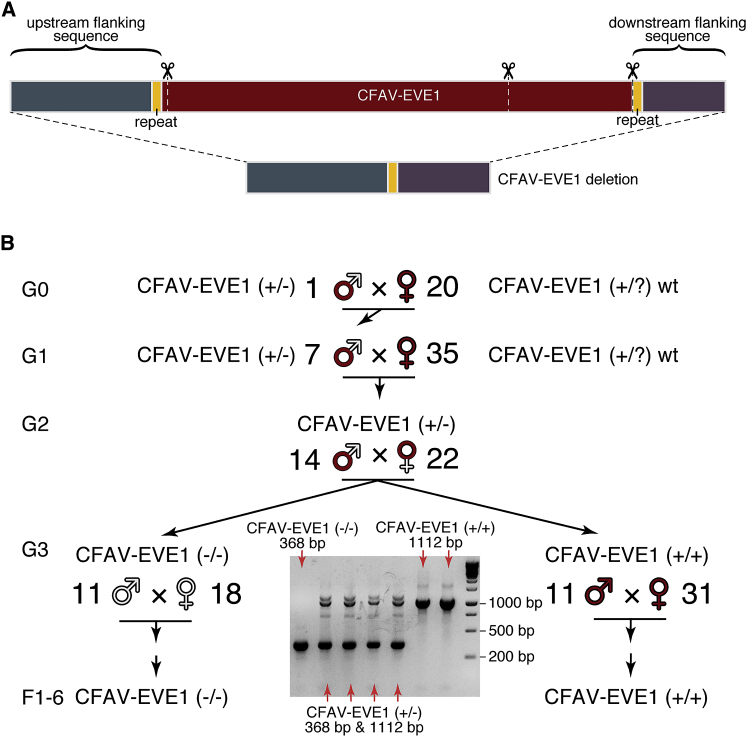
CRISPR-Cas9-Mediated Genome Editing of CFAV-EVE1 in *Aedes aegypti* (A) Deletion of the CFAV-EVE1 from the *Ae. aegypti* genome of the CFAV-free isofemale line using CRISPR-Cas9. The upper bar represents the CFAV-EVE1 with the flanking regions, and the three sgRNA target sites are shown with scissors. The lower bar represents the merged flanking regions without the CFAV-EVE1, where the short repeat sequences in the flanking regions (yellow segments on both bars) are merged into one. (B) Generation of the CFAV-EVE1 (+/+) and (−/−) *Ae. aegypti* lines after CRISPR-Cas9-mediated genome editing. A single G0 male mosquito heterozygous for the CFAV-EVE1 deletion (+/−) was outcrossed with wild-type females harboring the CFAV-EVE1. The resulting heterozygous male G1 progeny was outcrossed with wild-type females harboring the CFAV-EVE1. The G2 heterozygotes of both sexes were intercrossed to produce a mixed G3 progeny that was sorted into pure homozygous CFAV-EVE1 (+/+) and (−/−) lines. The letter G denotes the generation of mosquitoes originating from the CFAV-EVE1 heterozygous male and wild-type females. The letter F denotes the generation of the CFAV-EVE1 homozygous lines. The agarose gel picture represents a fraction of samples genotyped at G3, where the pure homozygous individuals were selected by PCR genotyping of a single leg. See also Table S1 and Table S3.

To directly test whether the presence of CFAV-EVE1 influences CFAV replication in *Ae. aegypti*, we used CRISPR-Cas9 genome editing to create a CFAV-EVE1 knockout (−/−) line and a homozygous CFAV-EVE1 control (+/+) line derived from our CFAV-free isofemale line. We designed two single-guide RNAs (sgRNAs) targeting the boundaries of CFAV-EVE1 and another sgRNA in the middle of CFAV-EVE1 to promote excision (Figure 3A; Table S3). The sgRNAs were injected together with recombinant Cas9 into mosquito embryos. We obtained a heterozygous male devoid of CFAV-EVE1 (Figure 3B) that was outcrossed with wild-type mosquitoes from the parental isofemale line for two consecutive generations. The progeny were carefully sorted into purely CFAV-EVE1 homozygous (+/+) and knockout (−/−) mosquitoes. Importantly, the CFAV-EVE1 (−/−) mosquitoes only included the genetically engineered deletion genotype and excluded individuals that could be naturally devoid of CFAV-EVE1.

### CFAV-Derived piRNA Production Is Strongly Reduced in the Absence of CFAV-EVE1

**Figure 4:**
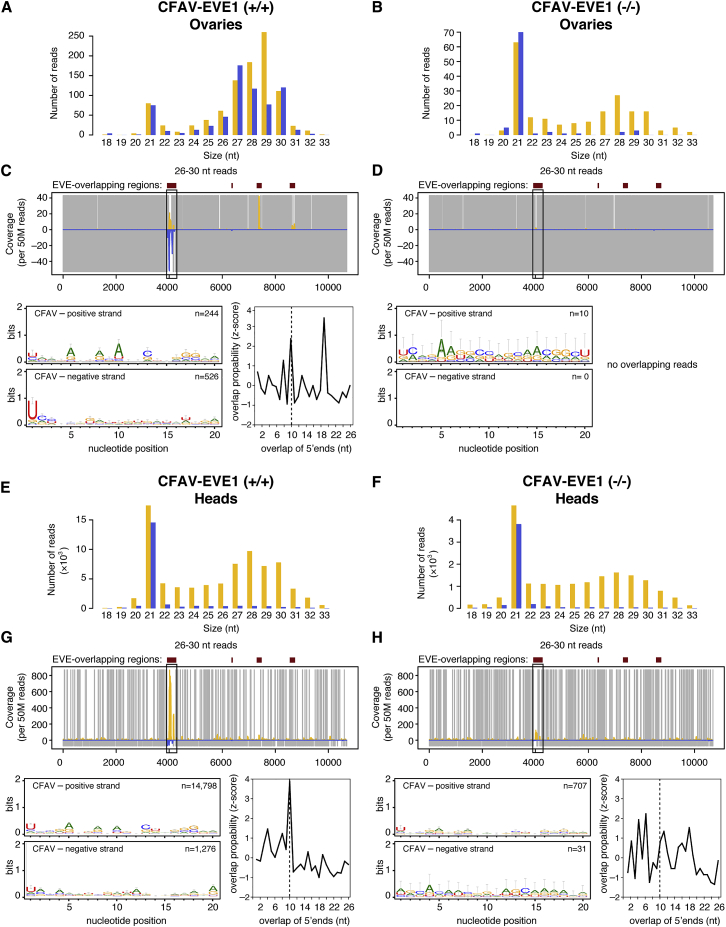
Ablation of CFAV-EVE1 Prevents CFAV-Derived piRNA Amplification (A, B, E, and F) Size distribution of sRNAs mapping to the CFAV genome in ovaries (A and B) and heads (E and F) from experimentally infected CFAV-EVE1 (+/+) (A and E) and CFAV-EVE1 (−/−) (B and F) mosquitoes 7 days post-injection. (C, D, G, and H) Analysis of CFAV-derived piRNAs in ovaries (C and D) and heads (G and H) from experimentally infected CFAV-EVE1 (+/+) (C and G) and CFAV-EVE1 (−/−) (D and H) mosquitoes 7 days post-injection. Mapping of 26- to 30-nt sRNAs (top), sequence logos of 26- to 30-nt sRNAs (bottom left), and overlap probability of 26- to 30-nt sRNAs (bottom right) is shown. Sequence logos and overlap probability were restricted to the NS2 region. In all panels, positive- and negative-sense reads with respect to the reference CFAV genome are shown in yellow and blue, respectively. Uncovered nucleotides are represented by gray lines. See also Figures S3, S4 and Table S5.

To determine whether the absence of CFAV-EVE1 affected the production of CFAV-derived piRNAs, we intrathoracically inoculated CFAV-EVE1 (+/+) and CFAV-EVE1 (−/−) mosquitoes with CFAV-KPP stock. 7 days post-infection, we dissected ovaries (germline tissue) and heads (somatic tissue) to prepare sRNA libraries from both tissues. Ovaries of mock-infected mosquitoes from the CFAV-EVE1 (+/+) line displayed the same sRNA profile (Figure S3A) as whole mosquitoes from the parental isofemale line (Figure 2C), with (−) piRNAs mainly derived from the NS2 region of CFAV-EVE1 and a 1U bias (Figures 2E and S3C). The heads of mock-infected mosquitoes (Figure S3E) contained few piRNAs mapping to the CFAV genome (<30 reads), consistent with the notion that germline tissues are the main producers of piRNAs [36]. As expected, mock-infected individuals from the CFAV-EVE1 (−/−) line did not harbor any piRNAs mapping to the CFAV genome in their ovaries and heads (Figures S3B, S3D, S3F, and S3H). This result confirmed that genome editing effectively removed the CFAV-EVE1 sequence and allowed us to test whether the absence of the EVE affected the production of virus-derived piRNAs upon experimental CFAV-KPP infection. Of note, we detected a small number of viral siRNAs mapping to the CFAV genome in mock-infected heads of the CFAV-EVE1 (+/+) line (86 reads) and the CFAV-EVE1 (−/−) line (15 reads). As these samples were run in the same flow cell that contained CFAV-infected samples (Figure 4) producing thousands of viral siRNA reads in head tissues (31,988 reads in the CFAV-EVE1 (+/+) line and 8,465 reads in the CFAV-EVE1 (−/−) line), the minute amount of viral siRNA detected in mock conditions is likely due to demultiplexing cross contamination, a common and recurrent problem in high-throughput sequencing of multiplexed samples [37].

Following CFAV-KPP inoculation, we detected abundant viral siRNAs in both CFAV-EVE1 (+/+) and CFAV-EVE1 (−/−) mosquitoes (Figures 4A and 4B). In addition, we detected virus-derived piRNAs and EVE-derived piRNAs with 1U and 10A bias and ping-pong amplification signature in the ovaries of CFAV-EVE1 (+/+) mosquitoes (Figure 4C). In contrast, (+) piRNAs mapping to the NS2 region of the CFAV-KPP genome were barely detectable in the ovaries of CFAV-EVE1 (−/−) mosquitoes (Figure 4D). Importantly, the detection of reads that unambiguously mapped to the virus showed that, even in the absence of the EVE, piRNAs were still produced from the virus genome upon infection (Figure S4A).

CFAV-KPP infection in the heads of CFAV-EVE1 (+/+) mosquitoes resulted in the production of CFAV-derived siRNAs as well as piRNAs (Figure 4E). The piRNAs corresponding to the NS2 region were in both sense and antisense orientation and presented a 1U-10A bias and 10-nt overlap of 5′ ends (Figure 4G). CFAV-KPP infection in the heads of CFAV-EVE1 (−/−) mosquitoes resulted in abundant CFAV-derived siRNAs (Figure 4F) and only piRNAs in sense orientation, without a ping-pong amplification signature, corresponding to primary piRNA production from the virus genome (Figures 4H and S4B).

Altogether, these results showed that the production of CFAV-derived piRNAs is profoundly modified in the absence of CFAV-EVE1. Production of primary piRNAs from CFAV-EVE1 is necessary to trigger the production of secondary virus-derived piRNAs from the virus genome. This observation suggests that piRNAs could have an antiviral activity in the joint presence of an EVE and its cognate virus.

### Increased CFAV Replication in Ovaries in the Absence of CFAV-EVE1

**Figure 5:**
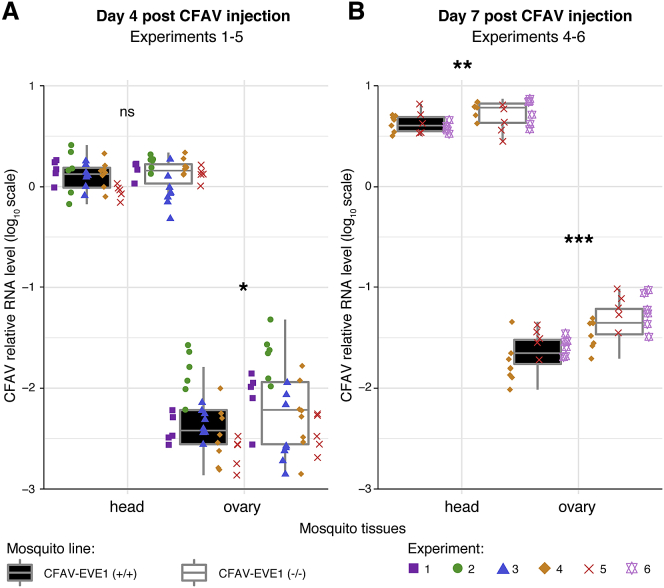
CFAV-EVE1 Ablation Results in Increased CFAV RNA Levels upon Viral Infection Relative CFAV RNA levels (normalized by the *rp49* housekeeping gene) in heads and ovaries of the CFAV-EVE1 (+/+) (black boxplot) and CFAV-EVE1 (−/−) (white boxplot) *Ae. aegypti* lines on day 4 (A) and day 7 (B) post-CFAV-inoculation. Data are shown for six separate experiments represented by color- and symbol-coded data points. Relative viral RNA loads are represented by boxplots in which the box denotes the median and interquartile range (IQR) and the whiskers extend to the highest and lowest outliers within 1.5 times the IQR from the upper and lower quartiles, respectively. Multivariate analysis of variance (MANOVA) was performed for each time point and tissue separately, accounting for the experiment, mosquito line, and interaction effects. Stars indicate statistical significance of the mosquito line main effect accounting for the experiment effect (^∗^p < 0.05; ^∗∗^p < 0.01; ^∗∗∗^p < 0.001; ns, not significant). The full MANOVA results are provided in Table S4. See also Table S1.

To assess the antiviral effect of piRNAs derived from the interaction between the EVE and the virus, we compared CFAV replication in CFAV-EVE1 (−/−) and CFAV-EVE1 (+/+) mosquitoes. To do so, we measured viral RNA levels in the heads and ovaries of females 4 and 7 days after CFAV inoculation. We performed six separate experiments using the same infectious dose and readout. The total amount of CFAV RNA produced by infected ovaries was significantly lower than the viral RNA produced in the heads (Figure 5). There was no consistent difference between mosquito lines across experiments for the CFAV RNA loads in heads collected on day 4 post-inoculation (Figure 5A; Table S4). Accounting for the inter-experiment variation, there was a significant difference of CFAV RNA loads in ovaries on day 4 post-inoculation, with CFAV replicating to higher levels in absence of the CFAV-EVE1 (Figure 5A; Table S4). On day 7 post-inoculation, CFAV RNA loads were significantly higher in mosquito heads (Figure 5B; Table S4) and even more so in mosquito ovaries (Figure 5B; Table S4) in the absence of the CFAV-EVE1. Together, these experiments showed that CFAV replicated to higher levels in the absence of CFAV-EVE1, most prominently in ovaries. These results demonstrate the antiviral activity of an EVE against its cognate virus.

## Discussion

It is well established that retroviral EVEs can play a role in host immunity (reviewed in [2]), most often as restriction factors [38, 39, 40, 41, 42] but also occasionally as cellular co-factors [43]. Whether this is the case for non-retroviral EVEs is still debated. Several studies attempted to prove that non-retroviral EVEs contribute to the immune antiviral response. Perhaps the best example is Borna disease virus (BDV) and its endogenous bornavirus-like element, which affects BDV polymerase activity and inhibits virus replication in a mammalian cell line when incorporated into the viral ribonucleoprotein [11]. Tassetto et al. observed that mosquito cells carrying an EVE related to CFAV were partially protected against a recombinant Sindbis virus engineered to contain the EVE sequence [44]. These *in vitro* experiments suggested that non-retroviral EVEs integrated in the host genome may provide antiviral protection against exogenous cognate viruses, but direct evidence from a natural system *in vivo* had not been provided until now.

The hypothesis that non-retroviral EVEs participate in antiviral immunity stems largely from accumulating evidence that they produce primary piRNAs [5, 6, 10, 12, 13, 20, 21]. The piRNA pathway is often referred to as the guardian of genome integrity because its canonical function is to silence TEs in the germline [45]. piRNA precursors are transcribed from genomic loci harboring transposon fragments that provide a genetic memory of past transposition invasion. The widespread occurrence of non-retroviral EVEs in *Aedes* mosquito genomes [6, 13] could reflect a similar mechanism whereby the function of EVEs would be to silence exogenous viruses with complementary sequences [17]. A major challenge to prove this hypothesis is that the viruses currently circulating generally do not share a high-nucleotide identity with the corresponding EVE sequences, preventing a possible match between EVE-derived piRNAs and the target viral RNA. In the present study, we overcame this obstacle by identifying a new EVE in *Ae. aegypti* mosquitoes from Thailand that is highly similar (∼96% nucleotide identity) to a contemporaneous CFAV strain. We used this naturally occurring insect-virus interaction to test the hypothesis that a non-retroviral EVE can inhibit virus replication via the piRNA pathway *in vivo*.

**Figure 6:**
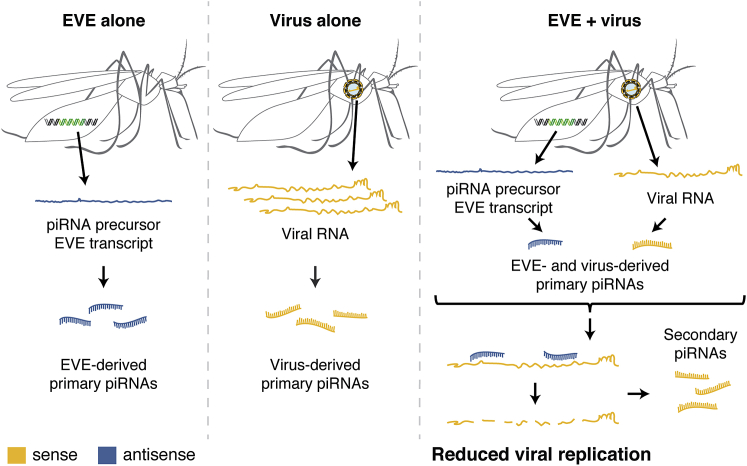
Model for the Antiviral Role of Non-retroviral EVEs in Mosquitoes Both a naturally occurring EVE (left panel) and exogenous viral infection (middle panel) produce primary piRNAs in antisense and sense orientation, respectively. Only when EVE and virus are present in the same mosquito do piRNAs acquire antiviral activity (right panel) through EVE-derived piRNAs targeting the viral genome. Under this model, integration of non-retroviral sequences into the host genome, their transcription into piRNA precursors, and their processing into antiviral piRNAs are mechanisms by which EVEs confer heritable, sequence-specific host immunity.

Our results revealed that, during both natural infection (mosquitoes carrying the CFAV EVE and naturally infected with CFAV) and controlled infection (mosquitoes carrying the CFAV EVE and experimentally inoculated with CFAV), the RNAs from the EVE and the virus interact through the piRNA pathway, resulting in inhibition of virus replication (Figure 6). Evidence of this interaction is provided by the abundant secondary piRNAs produced via the ping-pong amplification mechanism. Only when viral RNA is in presence of EVE-derived primary piRNAs does the piRNA pathway acquire its antiviral activity. Viral piRNAs alone are insufficient to induce this effect. Although viral piRNAs are commonly detected in mosquitoes [46], their antiviral function has remained equivocal [17]. Our study provides a clear demonstration that the piRNA pathway is involved in the mosquito antiviral response.

We observed that the piRNA-mediated antiviral effect of the CFAV EVE was strongest in the ovaries. Although recent research on arthropods suggests that protecting the germline was not necessarily its ancestral role [47], our results are consistent with a specialized role of non-retroviral, EVE-mediated antiviral immunity in germ cells. Presently, little is known about the pathogenicity of insect-specific viruses in mosquitoes in nature. However, because they are thought to be primarily transmitted vertically from mother to offspring, it is likely that insect-specific viruses reduce fecundity and/or fertility of their host. We speculate that the EVE-piRNA pathway combination may have evolved to control the replication of vertically transmitted viruses in the germline and maintain high fecundity and fertility. In fact, minimizing the detrimental effects of viral infection in the germline benefits both the host and the virus because the fitness of vertically transmitted viruses is directly linked to their host’s reproductive success [48, 49, 50].

Another open question is the degree of nucleotide identity required between the EVE and the virus for the antiviral activity to take place. Sequence mismatches reduce piRNA binding to its target sequences, and it was shown that more than three mismatches can effectively abolish piRNA recognition of the target sequence in *Drosophila* [51]. Even single mismatches in the seed sequence strongly reduce piRNA silencing efficiency in *Ae. aegypti* [52]. Therefore, viruses could escape EVE-mediated immunity by acquiring mutations, resulting in a possible coevolutionary arms race. Predicting the tempo and mode of such coevolutionary dynamics is difficult, even when the fitness cost of individual mutations is known [53]. Interestingly, in our study, the NS2 region of the CFAV EVE was most tightly involved in the interaction with the virus. This region corresponds to *fifo*, an open-reading frame (ORF) resulting from a ribosomal frameshift exclusively found in insect-specific flaviviruses [54]. The existence of two overlapping ORFs in this region (main frame and −1 frame) thus constrains sequence evolution. We speculate that this region may have been specifically retained as an EVE in the *Ae. aegypti* genome because the high level of purifying selection in the *fifo* region may prevent CFAV from escaping the antiviral mechanism by sequence divergence.

We observed that antiviral piRNAs against CFAV are produced in a similar manner in both naturally infected and virus-inoculated mosquitoes. Therefore, it is likely that EVEs represent a natural antiviral mechanism against vertically transmitted insect-specific viruses. Conversely, whether EVEs could contribute to suppress arbovirus transmission by mosquito vectors in nature is unlikely. Not only are arbovirus infections not prevalent (and rarely transmitted vertically) in natural mosquito populations, but they typically do not incur a fitness cost, presumably resulting in a lack of selective pressure for antiviral mechanisms against arboviruses [55]. This may help to explain why arbovirus-derived EVEs are uncommon in mosquito genomes [13]. Nevertheless, further exploration of the virome and genomes of wild mosquito populations, as well as additional experimental evidence in natural systems, are necessary to refine our understanding of the role of EVEs in mosquito antiviral immunity.

In view of our results and the increasing body of evidence from the literature [56], we conclude that EVEs constitute a universal system of heritable, sequence-specific antiviral immunity in eukaryotes, analogous to CRISPR-Cas immunity in prokaryotes. In the particular case of mosquitoes, integration of non-retroviral sequences into the host genome, their transcription within piRNA clusters, and their processing into antiviral sRNAs constitutes a mechanism by which these acquired viral sequences are co-opted to serve host immunity.

## STAR★Methods

### Key Resources Table

**Table:** 

REAGENT or RESOURCE	SOURCE	IDENTIFIER
**Bacterial and Virus Strains**		

CFAV-KPP	https://doi.org/10.1128/JVI.00705-19	ENA: LR596014

**Chemicals, Peptides, and Recombinant Proteins**		

RNase A/T1	Thermo Scientific	Cat# EN0551
DreamTaq Green DNA Polymerase	Thermo Scientific	Cat# EP0714
DNAzol DIRECT	Molecular Research Center, Inc.	Cat# DN131
NucleoSpin DNA Insect Kit	Machery-Nagel	Cat# 740470.50
NucleoSpin Tissue Kit	Machery-Nagel	Cat# 740952.50
MEGAscript T7 *in vitro* transcription kit	Thermo Scientific/Ambion	Cat# AM1333
Cas9 Nuclease, *S. pyogenes*	New England Biolabs	Cat# M0646
TRIzol Reagent	Thermo Scientific	Cat# 15596026
M-MLV Reverse Transcriptase	Thermo Scientific	Cat# 28025013
RNaseOUT Recombinant Ribonuclease Inhibitor	Thermo Scientific	Cat# 10777019
GoTaq qPCR Master Mix	Promega	Cat# A6002;Cat# TM318
TruSeq DNA PCR-free library preparation kit	Illumina	Cat# FC-121-3001
NEBNext Multiplex Small RNA Library Prep Set for Illumina (Set 1)	New England Biolabs	Cat# E7300
Universal miRNA Cloning Linker	New England Biolabs	Cat# S1315S

**Critical Commercial Assays**		

Qubit dsDNA HS (High Sensitivity) Assay Kit	Thermo Scientific	Cat# Q32851
Agilent Small RNA Kit	Agilent	Cat# 5067-1548

**Deposited Data**		

Outbred Thai *Aedes aegypti* with CFAV-EVE1 (+/?) and CFAV-EVE2 (+/?), full body, naturally infected with CFAV. sRNA.	This paper	HGW27BGXX, SRA: SAMN13244317
Outbred Thai *Aedes aegypti* with CFAV-EVE1 (+/?) and CFAV-EVE2 (+/?), full body, uninfected with CFAV. sRNA.	This paper	HG7CHBGXX, SRA: SAMN13244318
Thai *Aedes aegypti* isofemale line with CFAV-EVE1 (+/?), full body, CFAV IT injected, replicate 1. sRNA.	This paper	HVV5HBGXX, SRA: SAMN13244306
Thai *Aedes aegypti* isofemale line with CFAV-EVE1 (+/?), full body, CFAV IT injected, replicate 2. sRNA.	This paper	HVV5HBGXX, SRA: SAMN13244307
Thai *Aedes aegypti* isofemale line with CFAV-EVE1 (+/?), full body, CFAV IT injected, replicate 3. sRNA.	This paper	HVV5HBGXX, SRA: SAMN13244308
Thai *Aedes aegypti* isofemale line with CFAV-EVE1 (+/?), full body, mock IT injected, replicate 1. sRNA.	This paper	HVV5HBGXX, SRA: SAMN13244303
Thai *Aedes aegypti* isofemale line with CFAV-EVE1 (+/?), full body, mock IT injected, replicate 2. sRNA.	This paper	HVV5HBGXX, SRA: SAMN13244304
Thai *Aedes aegypti* isofemale line with CFAV-EVE1 (+/?), full body, mock IT injected, replicate 3. sRNA.	This paper	HVV5HBGXX, SRA: SAMN13244305
Isofemale line derived knockout line with CFAV-EVE1 (−/−), head, CFAV IT injected. sRNA.	This paper	HJ5CNBGX9, SRA: SAMN13244315
Isofemale line derived knockout sister line with CFAV-EVE1 (+/+), head, CFAV IT injected. sRNA.	This paper	HJ5CNBGX9, SRA: SAMN13244313
Isofemale line derived knockout line with CFAV-EVE1 (−/−), ovary, CFAV IT injected. sRNA.	This paper	HJ5CNBGX9, SRA: SAMN13244316
Isofemale line derived knockout sister line with CFAV-EVE1 (+/+), ovary, CFAV IT injected. sRNA.	This paper	HJ5CNBGX9, SRA: SAMN13244314
Isofemale line derived knockout line with CFAV-EVE1 (−/−), head, mock IT injected. sRNA.	This paper	HJ5CNBGX9, SRA: SAMN13244311
Isofemale line derived knockout sister line with CFAV-EVE1 (+/+), head, mock IT injected. sRNA.	This paper	HJ5CNBGX9, SRA: SAMN13244309
Isofemale line derived knockout line with CFAV-EVE1 (−/−), ovary, mock IT injected. sRNA.	This paper	HJ5CNBGX9, SRA: SAMN13244312
Isofemale line derived knockout sister line with CFAV-EVE1 (+/+), ovary, mock IT injected. sRNA.	This paper	HJ5CNBGX9, SRA: SAMN13244310
Thai *Aedes aegypti* isofemale line with CFAV-EVE1 (+/?). WGS.	This paper	SRA: SRR01437595
SRA accessions and BLAST search output of SRA search for CFAV related sequences	This paper, https://github.com/artembaidaliuk/SRA_search_CFAV_EVE_sequences/	N/A

**Experimental Models: Cell Lines**		

*Aedes albopictus* C6/36	ATCC	Cat# CRL-1660

**Experimental Models: Organisms/Strains**		

*Aedes aegypti* outbred colony	https://doi.org/10.1371/journal.pgen.1006111	N/A
*Aedes aegypti* isofemale line	https://doi.org/10.1371/journal.pgen.1006111	Line D
	https://doi.org/10.1371/journal.pgen.1003621	
Isofemale line derived knockout line with CFAV-EVE1 (−/−)	This paper	N/A
Isofemale line derived knockout sister line with CFAV-EVE1 (+/+)	This paper	N/A

**Oligonucleotides**		

All primer sequences are listed in Table S1	[57]	N/A
Repair template sequence is listed in Table S3		N/A

**Software and Algorithms**		

BLAST	[60]	N/A
SRA Toolkit v2.9.6	https://doi.org/10.1093/nar/gkq1019https://github.com/ncbi/sra-tools	N/A
metaSPAdes v3.11.0	https://doi.org/10.1101/gr.213959.116	N/A
R v3.5.2	http://www.r-project.org/	N/A
Trimmomatic v0.36	https://doi.org/10.1093/bioinformatics/btu170	N/A
Bowtie2 v2.3.4.3	https://doi.org/10.1038/nmeth.1923	N/A
Cutadapt v1.18	https://doi.org/10.14806/ej.17.1.200	N/A
Ray v2.3.1-mpi	https://doi.org/10.1089/cmb.2009.0238	N/A
Geneious v10.2.3	https://www.geneious.com	N/A
bedtools v2.25.0	https://doi.org/10.1093/bioinformatics/btq033	N/A
LoFreq v2.1.3.1	https://doi.org/10.1093/nar/gks918	N/A
CRISPOR	http://crispor.tefor.net/	N/A
FastQC v0.10.1	http://www.bioinformatics.babraham.ac.uk/projects/fastqc/	N/A
Bowtie1 v1.1.2	https://doi.org/10.1186/gb-2009-10-3-r25	N/A
Galaxy	https://doi.org/10.1093/nar/gky379	N/A
WebLogo 3	https://doi.org/10.1101/gr.849004	N/A
Graphpad Prism 6	https://www.graphpad.com/scientific-software/prism/	N/A
tidyverse v1.3.0 (R package)	https://doi.org/10.21105/joss.01686 [58]	N/A
viridis v0.5.1 (R package)	https://CRAN.R-project.org/package=viridis	N/A
parsedate v1.2.0 (R package)	https://CRAN.R-project.org/package=parsedate	N/A
Rsamtools v1.20.4 (R package)	https://doi.org/10.18129/B9.bioc.Rsamtools	N/A
ShortRead v1.26.0 (R package)	https://doi.org/10.18129/B9.bioc.ShortRead [59]	

**Other**		

Mississippi, Galaxy	https://mississippi.snv.jussieu.fr/	N/A
SRA_blast.sh	This paper, https://github.com/artembaidaliuk/SRA_search_CFAV_EVE_sequences/	N/A
script-SRA_prefetch_into_custom_dir.sh	This paper, https://github.com/artembaidaliuk/SRA_search_CFAV_EVE_sequences/	N/A

### Resource Availability

#### Lead Contact

Further information and requests for resources and reagents should be directed to and will be fulfilled by the Lead Contact, Maria-Carla Saleh (carla.saleh@pasteur.fr)

#### Material Availability

Research materials generated in this study are available upon request.

#### Data and Code Availability Statement

The genome sequence of the *Aedes aegypti* isofemale line is available at Sequence Read Archive (SRA): SRR01437595. All sRNA sequencing data are available at SRA: PRJNA588447. The code and data of the SRA survey were deposited to a public repository (https://github.com/artembaidaliuk/SRA_search_CFAV_EVE_sequences).

### Experimental Model and Subject Details

#### Mosquito origin and maintenance

An outbred laboratory colony of *Ae. aegypti* mosquitoes originally sampled in 2013 from a wild population in Thep Na Korn Village, Kamphaeng Phet Province, Thailand [35] was found to be infected with CFAV [32] and was used in this study for CFAV-EVE1 and CFAV-EVE2 detection by gDNA PCR and sRNA sequencing of naturally infected and uninfected mosquitoes. An isofemale line of *Ae. aegypti* originating from Kamphaeng Phet Province, Thailand was used for experimental infections *in vivo*. The isofemale line was created in 2010 as the progeny of a single-pair mating between a wild male from Mae Na Ree village and a wild female from Nhong Ping Kai village [34, 35]. The inability to isolate CFAV from mosquito homogenates on C6/36 (*Ae. albopictus*) cells (ATCC CRL-1660) and to detect CFAV by RT-PCR directly on mosquito RNA confirmed that the isofemale line was CFAV-free. Mosquitoes were maintained under standard insectary conditions (27°C, 70% relative humidity and 12h:12h light:dark cycle). Larvae were reared in plastic trays filled with 1.5 L of dechlorinated tap water at a density of 200 larvae per tray and provided with 200 mg of TetraMin fish food (Tetra) on days 0 and 2 and 400 mg on day 4. After emergence, adult mosquitoes were housed in plastic cages under standard insectary conditions (27°C, 70% relative humidity and 12h:12h light:dark cycle) and provided with 10% sucrose solution *ad libitum*.

### Method Details

#### Ethics statement

Genetic modification of *Ae. aegypti* was performed under authorization number 4018 (bis) from the French Ministry of Higher Education, Research and Innovation.

#### Survey of CFAV-related EVEs in public sequencing data of *Aedes aegypti*

The accession numbers for the *Ae. aegypti* sequencing data were selected using the web platform of the SRA database [61]. We used BLAST (megablast) search [60] implemented in the SRA Toolkit [61] to search for CFAV-like sequences in the preselected SRA data. The BLAST search resulted in 796 RNA-seq and 709 WGS runs tested, released before January 30 and February 6, 2020, respectively. Full-genome CFAV sequences from Thailand CFAV-Bangkok (European Nucleotide Archive (ENA): LR694074) [62] and CFAV-KPP (ENA: LR596014) [32] were used as queries. Visualization of positive hits was performed in R v3.6.1 (http://www.r-project.org/). Using the online BLAST tool (megablast), the CFAV-EVE1 sequence was detected in the supercontig 1.109 of the AaegL3 genome assembly (GenBank: GCA_000004015.3) but absent from the AaegL5 genome assembly (GenBank: GCA_002204515.1). The CFAV-EVE1 sequence was reconstructed from a published WGS dataset (SRA: SRR5562867) using metaSPAdes v3.11.0 [63]. Reads from the WGS dataset were first quality trimmed with Trimmomatic v0.36 [64] and aligned against the AaegL5 genome assembly with Bowtie2 v2.3.4.3 (–end-to-end–very-fast) [65] to filter out all non-EVE reads. The CFAV-EVE2 sequence was reconstructed from SRA: SAMN04480331, SAMN04480332, SAMN04480333. Reads were trimmed with Cutadapt v1.18 [66]. Relaxed local Bowtie2 v2.3.4.3 alignment (–local -D 20 -R 3 -L 11 -N 1–gbar 1–mp 3) was used in order to preselect CFAV-derived reads, which were then used for *de novo* assembly with Ray v2.3.1-mpi tool [67]. The contigs obtained from all three SRA samples were combined into a single sequence of CFAV-EVE2 using Geneious v10.2.3 software (https://www.geneious.com). The sequence was then verified by Bowtie2 alignment (–local) of the reads, coverage and single nucleotide variant calculation by bedtools v2.25.0 and LoFreq v2.1.3.1, respectively [68, 69]. Both CFAV-EVE1 and CFAV-EVE2 sequences with annotations are available in Table S2.

#### Live *Aedes aegypti* mosquitoes

##### Whole-genome sequencing of the isofemale line

The whole genome of the *Ae. aegypti* isofemale line was sequenced at the 20^th^ generation of colonization. The DNA was extracted from a total of 144 virgin females following a published method [70]. Six pools of 4 mosquitoes were homogenized in 240 μL of the following buffer: 0.1 M NaCl, 0.2 M sucrose, 0.1 M Tris buffer, 0.05 M EDTA, 0.5% SDS, pH adjusted to 9.2 with NaOH. The homogenates were incubated at 65°C for at least 35 min and 34 μL of 8 M KAc were added to the heated homogenates and cooled on ice for 30 min. Supernatants were transferred to new tubes, mixed with an equal volume of 100% ethanol and incubated for 5 min at room temperature (20-25°C). The DNA was pelleted by 15-min centrifugation at 21,100*g* and washed with 75% ethanol. The pellet was resuspended in 100 μL of PCR-grade water. This procedure was repeated 6 times and DNA elutes from all pools were gathered in a single tube and precipitated by adding 1/10 of 3M NaAc and 2.5x of cold 100% ethanol, followed by a washing step with 75% ethanol. The final elution was done in 400 μL of PCR-grade water. The genomic DNA was treated with RNase A/T1 (Thermo Scientific) for 30 min at 37°C and precipitated with NaAc again. The quality of the resulting DNA was assessed by Nanodrop (Thermo Scientific), Qubit HS DNA Assay Kit (Invitrogen), and 1% agarose gel migration. The DNA sequencing was performed commercially by Macrogen Europe (http://www.macrogen.com). A TruSeq PCR-free DNA shotgun library (550-bp inserts) was sequenced on an Illumina HiSeq 4000 platform (2 × 100 bp). The genome sequence of the isofemale line was deposited to SRA: SRR01437595.

##### DNA extraction and CFAV-EVE1-specific and CFAV-EVE2-specific PCRs

To verify the presence and prevalence of the CFAV-EVE1 in the *Ae. aegypti* isofemale line and outbred colony, DNA was extracted by two different methods. Genomic DNA was extracted from single legs of individual mosquitoes or whole individual mosquitoes using NucleoSpin DNA Insect Kit (Machery-Nagel) or NucleoSpin Tissue Kit (Macherey-Nagel) following the manufacturers’ instructions. Final elution was performed with 20 μL of the elution buffer. The DNA was used as a template for CFAV-EVE1-specific qualitative PCR with DreamTaq Green DNA Polymerase (Thermo Scientific) following the manufacturer’s recommendations, and using S7, EVE-GT-external, EVE-GTlong-external and/or EVE-GT-internal primers (Table S1). The CFAV-EVE2 sequence was detected with the CFAV-EVE2 primer set (Table S1).

Alternatively, DNAzol DIRECT (Molecular Research Center, Inc.) was used following manufacturer’s instructions, where DNA was extracted from single legs by placing a leg in 200 μL of DNAzol DIRECT in a 1.5-mL screw-cap tube partially filled with glass beads and homogenized. The lysate was centrifuged 15-30 s at 21,100*g* and incubated at room temperature (20-25°C) for at least 20 min. Subsequently, 0.5-1 μL of lysate was used directly into a 20-μl PCR reaction. The same DNAzol DIRECT extraction procedure was used for whole mosquitoes, but the lysate was diluted 1:50 in PCR-grade water and 0.5-1 μL of the dilution was used in a 20 μL PCR reaction as described above.

#### CRISPR/Cas9-mediated genome engineering

##### SgRNA design and synthesis

The *Ae. aegypti* isofemale line containing the CFAV-EVE1 was used to produce pure homozygous CFAV-EVE1 (+/+) and (−/−) lines using CRISPR/Cas9 as previously described for *Ae. aegypti* [71]. The single-guide RNAs (sgRNAs) were designed using CRISPOR (http://crispor.tefor.net/) by searching for 20-bp sgRNAs with the NGG protospacer-adjacent-motif (PAM). In order to reduce chances of off-target mutations, only sgRNAs with off-target sites which contained three or more mismatches were selected. Two sgRNAs with cut-sites proximal to the boundaries of the CFAV-EVE1 were chosen in order to delete the full CFAV-EVE1 sequence. A third sgRNA in the middle of the EVE sequence was added to facilitate deletion of the CFAV-EVE1 sequence. sgRNA sequences with their most probable off-target sites are represented in Table S3. SgRNAs were produced as previously described [71]. Double-stranded DNA templates for each sgRNA were produced by template-free PCR with two partially overlapping oligos (PAGE-purified, Sigma-Aldrich). Where necessary, one or two guanines were added to the 5′ end of the guide sequence within the primer to ensure the format “5′-GG(N18-20)-3′” in order to facilitate *in vitro* transcription with MEGAscript T7 *in vitro* transcription kit (Ambion). Transcribed sgRNAs were purified with MEGAclear kit (Invitrogen). Quality of sgRNAs were assessed with Bioanalyzer, Agilent 2100 Small RNA kit (Agilent).

##### Repair template design

We designed a 110-nt repair template with homology arms (HA) to the upstream and downstream flanking regions of the CFAV-EVE1 and extending to the sgRNA cut-sites (3 bp upstream of the PAM). The annotated sequence of the repair template is provided in Table S3. Due to the 5′ sgRNA having a cut-site inside the CFAV-EVE1 sequence, mismatches were artificially incorporated into to the 5′ HA of the repair template to ensure disruption of the CFAV-EVE1 sequence while maintaining enough homology to facilitate homologous recombination and deletion of CFAV-EVE1. An sgRNA sequence (with PAM) exogenous to the *Ae. aegypti* genome was also included in the repair template in an attempt to incorporate this guide sequence for further CRISPR/Cas9-mediated mutagenesis of this site. However, this and the modified CFAV-EVE1 sequences ultimately failed to get incorporated in CFAV-EVE1 (−/−) line genome. This could be explained by the presence of the 5′-TAAAAGTGGCGACGAG-3′ sequence contained in each flanking region of the CFAV-EVE1 that might have mediated the homology-dependent double-strand break repair independently of the repair template or that one homology arm acted as a truncated repair template.

##### Egg microinjection

The final microinjection mix contained 322 ng/μL spCas9 protein (New England Biolabs) with 40 ng/μL of each sgRNA and 127 ng/μL of the ssDNA repair template. The microinjection of *Ae. aegypti* embryos was performed according to standard protocols [72]. *Ae*. *aegypti* females were engorged with commercial rabbit blood (BCL) via an artificial membrane feeding system (Hemotek). At least 3 days post blood meal, females were transferred into egg-laying vials and oviposition was induced by placing mosquitoes into dark conditions. Embryos were injected 30-60 min post oviposition. Embryos were hatched by being placed in water at least 3 days post injection and reared to adult stage as described above under mosquito maintenance. The generation 0 (G0) virgin adult mosquitoes were genotyped using a single leg DNA by PCR with EVE-GTlong-external primers (Table S1). The deletion in the CFAV-EVE1 heterozygous PCR products was confirmed by Sanger sequencing.

##### Generation of the CFAV-EVE1 (+/+) and (−/−) lines

A single male mosquito (G0) with a verified CFAV-EVE1 heterozygous genotype was mated with 20 wild-type females. The progeny (G1) were genotyped and 7 heterozygous males were mated with 35 wild-type females. G2 progeny were genotyped and 14 heterozygous males were mated with 22 heterozygous females. G3 progeny were genotyped and pure CFAV-EVE1 (+/+) and CFAV-EVE1 (−/−) lines were created by pooling homozygous positive (11 males and 31 females) and negative (11 males and 18 females) mosquitoes, respectively. The progeny of these crosses (F1) were verified by the PCR with CFAV-EVE1 external primers in 3 pools of 20 mosquitoes from each line. The lines were reared according to the standard rearing procedures described above. Further line genotype verification was performed at F3, F4, and F5. The F4 generation of mosquitoes was used for sRNA sequencing, which confirmed the almost complete absence of sRNAs complementary to CFAV in the CFAV-EVE1 (−/−) line, hence, the purity of the CFAV-EVE1 deletion and the absence of any other CFAV-related EVE that could have produced sRNAs.

#### CFAV experimental infections *in vivo*

##### CFAV isolate and injection conditions

A wild-type CFAV strain (CFAV-KPP; ENA: LR596014) previously isolated from the *Ae. aegypti* outbred laboratory colony [32] was used for experimental infections of the CFAV-free *Ae. aegypti* isofemale line and the genetically modified lines 3-7 days post emergence. To prepare CFAV-KPP stocks, the cell-culture supernatant of infected C6/36 *Aedes albopictus* cells was harvested after 7 days of virus amplification and stored at −80°C as previously described [32]. Initially, CFAV-KPP stocks were obtained directly from mosquito homogenates inoculated onto C6/36 cells followed by three amplification passages. Later CFAV-KPP stocks were obtained by genomic RNA transfection of C6/36 cells followed by two amplification passages as previously described [32]. The infectious titer of the virus stocks was measured by 50% tissue-culture infectious dose (TCID_50_) assay as previously described [32]. The estimated titers of the CFAV-KPP stocks from mosquito homogenates and from the genomic RNA template were 1.14 × 10^7^ TCID_50_ units/ml and 7.91 × 10^5^ TCID_50_ units/ml, respectively. The first intrathoracic injection of the *Ae. aegypti* isofemale line harboring the CFAV-EVE1 was done with the initial CFAV-KPP stock made from mosquito homogenates. Female mosquitoes were each injected with 786 TCID_50_ units each using Nanoject II Auto-Nanoliter Injector (Drummond), then sacrificed on day 7 post injection and pooled RNA from 10 whole bodies was used for the first sRNA library preparation and sequencing. Mock injections were performed with naive C6/36 cell-culture supernatant. Experimental infections of CFAV-EVE1 (+/+) and (−/−) lines (referred to as experiments 1-6) were performed with CFAV-KPP stocks produced from the genomic RNA template. Female mosquitoes were each intrathoracically injected with 50 TCID_50_ units in experiments 1-6 using Nanoject III Programmable Nanoliter Injector (Drummond). In experiment 5, mock injection was done with the naive C6/36 cell-culture supernatant. RNA from the pools of heads and ovaries of injected females dissected on day 4 (experiments 1-5) or on day 7 (experiments 4-6) was used for the RT-qPCR with CFAV-specific primers and additionally for sRNA sequencing (experiment 5, day 7). In experiment 1, RNA was extracted from 5 pools of 4 tissues (pairs of ovaries or heads in all 6 experiments) per condition (mosquito line). In experiment 2, RNA was extracted from 6 pools of 5 tissues per condition (mosquito line). In experiment 3, RNA was extracted from 8 pools of 4 tissues per condition (mosquito line). In experiment 4, RNA was extracted from 6-8 pools of 4 tissues per condition (mosquito line and day post injection). In experiment 5, RNA was extracted from 5 pools of 9 tissues per condition (mosquito line and day post injection). Finally, in experiment 6, RNA was extracted from 5 pools of 5 tissues per condition (mosquito line). Mosquitoes were from generation F3 in experiments 1-4, generation F4 in experiment 5, and generation F6 in experiment 6.

##### CFAV RNA quantification

Total RNA was extracted and purified from mosquito tissues using TRIzol Reagent (Invitrogen) following manufacturer’s instructions with RNA elution in 30 μL of PCR-grade water. cDNA synthesis was performed using M-MLV reverse transcriptase (Invitrogen) by mixing 10 μL of eluted RNA with 100 ng of random primers (Roche), 10 nmol of each dNTP, 2 μL of DTT, 4 μL of 5X First-Strand Buffer, 0.5 μL of PCR-grade water, 20 units of RNaseOUT recombinant ribonuclease inhibitor (Invitrogen), and 200 units of M-MLV reverse transcriptase in a final reaction volume of 20 μL. Reactions were incubated for 10 min at 25°C, 50 min at 37°C, 15 min at 70°C, and held at 4°C until further use or stored at −20°C. cDNA was diluted 1:5 before quantitative analysis by qPCR was done using GoTaq qPCR Master Mix (Promega) following manufacturer’s recommendations. Primer sequences are provided in Table S1. CFAV qPCR values were normalized by the housekeeping gene *rp49* qPCR values.

#### Small-RNA sequencing

##### sRNA library preparation and sequencing

Total RNA from pools of 5 to 10 mosquitoes was subjected to acrylamide gel (15% acrylamide/bisacrylamie, 37.5:1, and 7M urea) electrophoresis to purify sRNAs of 19-33 nt in length. Purified sRNAs were used for library preparation with NEBNext Multiplex Small RNA Library Prep Set for Illumina (New England Biolabs) with 3′ adaptor, Universal miRNA Cloning Linker – S1315S (New England Biolabs) and in-house designed indexed primers. Libraries were diluted to 4 nM and sequenced on a NextSeq 500 sequencer (Illumina) with a NextSeq 500 High-Output Kit v2 (Illumina) (52 cycles).

##### Analyses of small-RNA sequencing data

The quality of the fastq files was assessed with FastQC software (http://www.bioinformatics.babraham.ac.uk/projects/fastqc/). Low-quality bases and adaptors were trimmed from each read using Cutadapt. Only reads with an acceptable quality (Phred score > 20) and the adaptor sequence at the 5′ end were retained. A second set of graphics was generated by the FastQC software using the fastq files trimmed using Cutadapt. Reads were mapped to target sequences using Bowtie1 [73] (one mismatch allowed between the read and its target for initial mapping or no mismatch allowed for target-specific mapping) or the Bowtie2 tool with default options for the sRNA or DNA library, respectively. The Bowtie1 tool (sRNA library) and the Bowtie2 tool (DNA library) generate results in sequence alignment/map (SAM) format. All SAM files were analyzed by the SAMtools package to produce bam indexed files. Homemade R scripts with Rsamtools and Shortreads in Bioconductor software were used for analysis of the bam files. For the analysis of sequence logos and sRNA overlaps, sRNA reads aligned to the CFAV-EVE1 sequence or to the CFAV genomic RNA were processed in Galaxy [74]. To generate sequence logos, reads of 26-30 nt in length were filtered and separated according to their genomic orientation. The selected reads were converted into FastA format, trimmed at the 3′ end to 20 nt and converted to RNA letters using the corresponding FastA/FastQ tools. The processed reads were used as input for the Weblogo tool available in the Galaxy toolshed [75]. For the analysis of ping-pong signatures, aligned reads were loaded into the Mississippi instance of Galaxy (https://mississippi.snv.jussieu.fr/). SAM files containing the reads of 26-30 nt in length were used as input for the Small RNA signatures tool. The Z-scores of the calculated overlap probabilities were plotted with Graphpad Prism. All sRNA sequencing library sizes with the number of CFAV-mapped reads are reported in Table S5. All data are available at SRA: PRJNA588447.

### Quantification and Statistical Analysis

To compare CFAV relative RNA levels between CFAV-EVE1 (+/+) and (−/−) mosquito lines, pools of mosquito tissues were considered as biological units of replication. Normalized CFAV RNA levels were log_10_-transformed. Type III multivariate analysis of variance (MANOVA) was performed separately for each time point (day 4 and day 7 post injection) and each tissue type (heads and ovaries). The linear model included experiment, mosquito line, and their interaction as covariates. The interaction term was removed from the model when its effect was statistically non-significant (p > 0.05), and type II MANOVA was performed instead. Statistical analyses were performed in R v3.5.2 (http://www.r-project.org/).

## Supplemental Information

Document S1. Figures S1–S4 and Tables S1–S5

Data S1. BLAST Alignment of Publicly Available *Aedes aegypti* Sequencing Data to the CFAV Genome, Related to Figure 1A Each line represents a BLAST hit from a Sequence Read Archive (SRA) RNA-seq sample, an SRA whole-genome sequencing (WGS) sample, or the AeagL3 assembly (indicated by an asterisk). BLAST hits are aligned to the CFAV genome and ordered according to the pattern of coverage. The percentage of nucleotide identity to the CFAV-Bangkok (RNA) or CFAV-KPP (DNA) genomes is indicated by the color gradient shown at the bottom of the plot. The sample names colored in red and blue were used to reconstruct the sequence of CFAV-EVE1 and CFAV-EVE2, respectively.

Document S2. Article plus Supplemental Information
