# Supplementary material for: Non-retroviral Endogenous Viral Element Limits Cognate Virus Replication in Aedes aegypti Ovaries
Source: Curr Biol. 2020 Sep 21;30(18):3495–3506.e6. doi: 10.1016/j.cub.2020.06.057 (PMC7522710; doi:10.1016/j.cub.2020.06.057)
Supplement: Document S1. Figures S1–S4 and Tables S1–S5 [file mmc1.pdf]

Current Biology, Volume *30*

Supplemental Information

Non-retroviral Endogenous Viral Element Limits Cognate Virus Replication in *Aedes aegypti* Ovaries

Yasutsugu Suzuki, Artem Baidaliuk, Pascal Miesen, Lionel Frangeul, Anna B. Crist, Sarah H. Merkling, Albin Fontaine, Sebastian Lequime, Isabelle Moltini-Conclois, Hervé Blanc, Ronald P. van Rij, Louis Lambrechts, and Maria-Carla Saleh

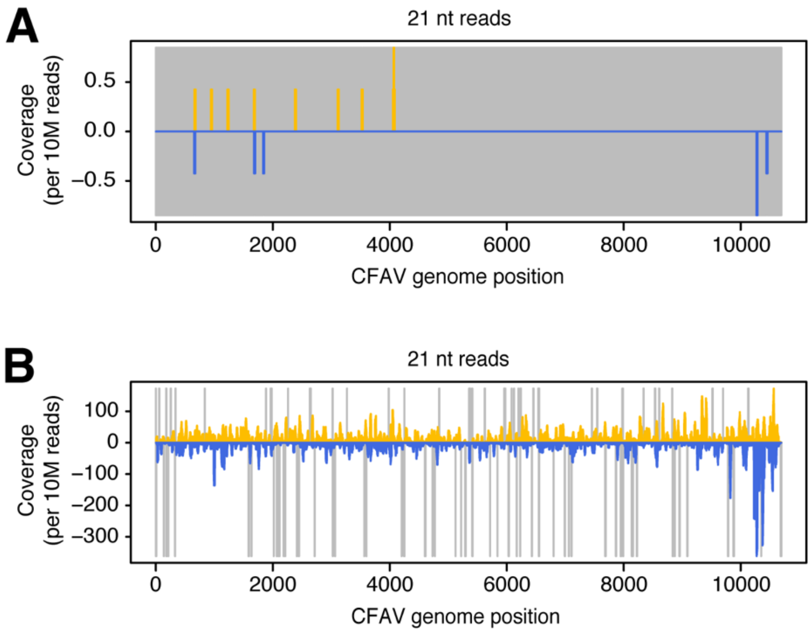

Figure S1. siRNA response to a natural CFAV infection in *Aedes aegypti* mosquitoes harboring CFAV-EVE1 and CFAV-EVE2. Related to Figure 1.

Profiles of siRNAs mapping to the CFAV-KPP genome from naturally CFAV-uninfected (**A**) or CFAV-infected (**B**) mosquitoes from the outbred colony. Positive- and negative-sense reads are shown in yellow and blue, respectively. Uncovered nucleotides are represented by gray lines.

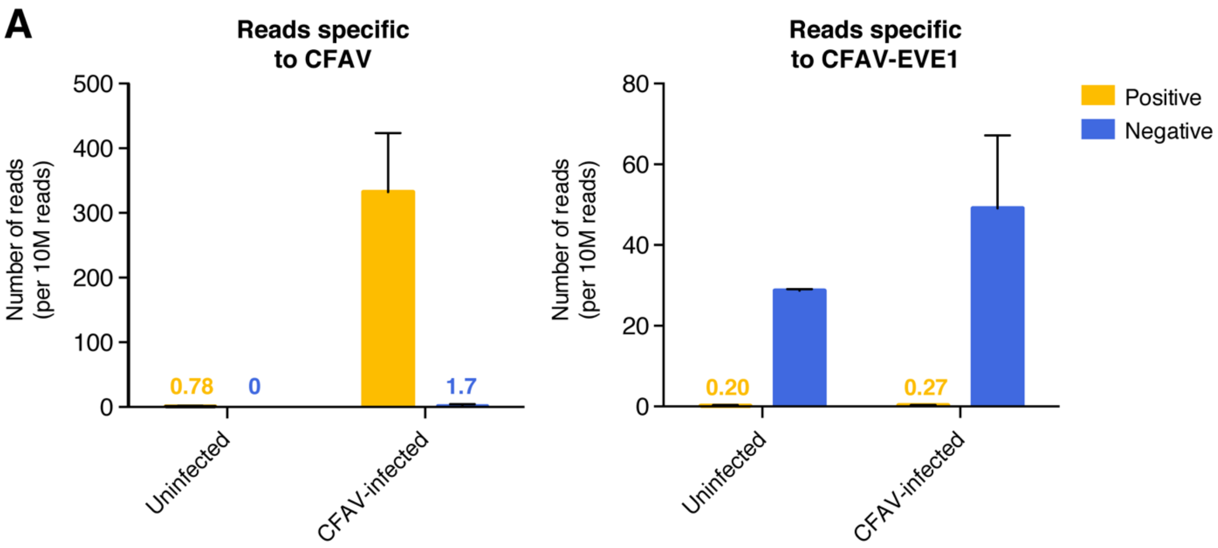

Figure S2. Target specificity of piRNAs upon CFAV infection in the *Aedes aegypti* isofemale line harboring CFAV-EVE1. Related to Figure 2.

Number of 26-30 nt sRNA reads unambiguously mapping to the CFAV-KPP genome (**A**) or to the CFAV-EVE1 locus (**B**) in uninfected or CFAV-infected individuals of the isofemale line. Yellow and blue colors represent positive-sense and negative-sense reads, respectively. For visual clarity, the normalized number of reads is shown above the bars when the number of reads is <1% of the maximum value.

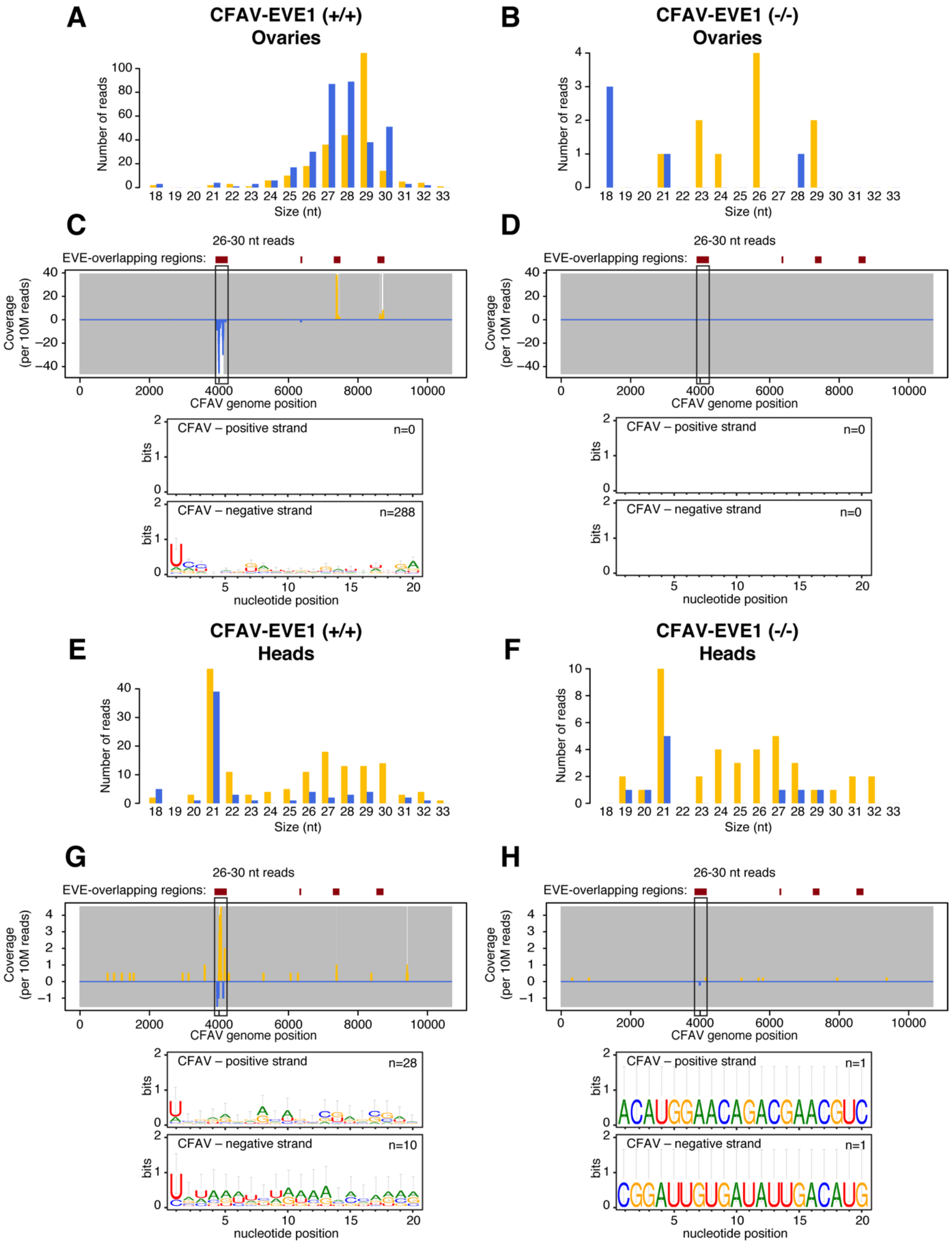

Figure S3. Small RNA profiles of CFAV-EVE1 (+/+) and (-/-) mock-inoculated *Aedes aegypti* lines. Related to Figure 4.

Size distribution of sRNAs mapping to the CFAV genome in ovaries (**A-B**) and heads (**E-F**) from experimentally mock-infected CFAV-EVE1 (+/+) (**A,E**) and CFAV-EVE1 (-/-) (**B,F**) mosquitoes 7 days post injection. Analysis of CFAV-derived piRNAs in ovaries (**C-D**) and heads (**G-H**) from experimentally mock-infected CFAV-EVE1 (+/+) (**C,G**) and CFAV-EVE1 (-/-) (**D,H**) mosquitoes 7 days post injection. Mapping (top) and sequence logos (bottom) of 26-30 nt sRNAs. Positive- and negative-sense reads with respect to the reference CFAV genome are shown in yellow and blue, respectively. Uncovered nucleotides are represented by gray lines.

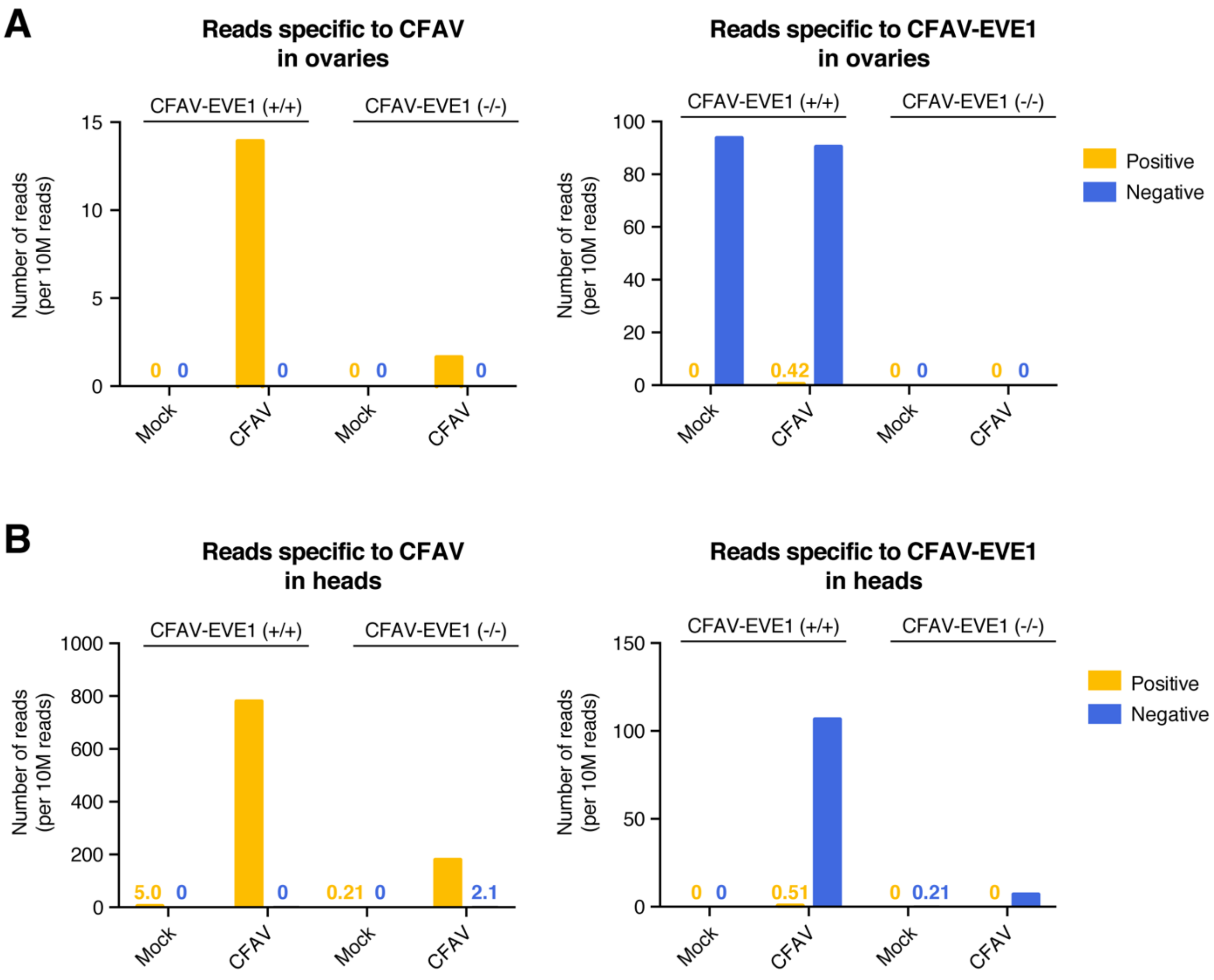

Figure S4. Target specificity of the piRNAs upon CFAV infection in CFAV-EVE1 (+/+) and (-/-) *Aedes aegypti* lines. Related to Figure 4.

Number of 26-30 nt sRNA reads unambiguously mapping to the CFAV-KPP genome (left) or to the CFAV-EVE1 locus (right) in ovaries (**A**) and heads (**B**) of mosquitoes from the CFAV-EVE1 (+/+) line and CFAV-EVE1 (-/-) line 7 days after mock-infection or CFAV infection. Yellow and blue colors represent positive-sense and negative-sense reads, respectively. For visual clarity, the normalized number of reads is shown above the bars when the number of reads is <1% of the maximum value.

Name	Genomic region	Application	Direction	Primer sequence (5'->3')	Length of PCR product	Annealing temperature
CFAV-NS3q-F	CFAV NS3	quantification	forward	ACACGAGTGAAGCTGGTTGA	92 bp	56°C
CFAV-NS3q-R	CFAV NS3	quantification	reverse	ACATACGTTCCTGGTTCCCG	92 bp	56°C
RP49q-F	*Aedes aegypti rp49*	houskeeping gene quantification	forward	ACAAGCTTGCCCCCAACT	97 bp	60°C
RP49q-R	*Aedes aegypti rp49*	houskeeping gene quantification	reverse	CCGTAACCGATGTTTGGC	97 bp	60°C
S7-F	*Aedes aegypti rps7*	houskeeping gene detection	forward	GGGACAAATCGGCCAGGCTATC	292 bp, cDNA; 406 gDNA	64°C
S7-R	*Aedes aegypti rps7*	houskeeping gene detection	reverse	TCGTGGACGCTTCTGCTTGTTG	292 bp, cDNA; 406 gDNA	64°C
EVE-GT-external-F	*Aedes aegypti* CFAV-EVE1 upstream flanking region	detection and genotyping	forward	GGTCGAAGCGAGATGAACTGTG	1090 bp	70°C
EVE-GT-external-R	*Aedes aegypti* CFAV-EVE1 downstream flanking region	detection and genotyping	reverse	GGCCAAATGCTGCTGCGAG	1090 bp	70°C
EVE-GTlong-external-F	*Aedes aegypti* CFAV-EVE1 upstream flanking region	detection and genotyping	forward	CTTATGTAGTAGCTACAGGTCGAAGCGAG	1112 bp	70°C
EVE-GTlong-external-R	*Aedes aegypti* CFAV-EVE1 downstream flanking region	detection and genotyping	reverse	GCAATGGCCAAATGCTGCTGCGAG	1112 bp	70°C
EVE-GT-internal-F	*Aedes aegypti* CFAV-EVE1 NS5 region	detection and genotyping	forward	CACATCCCTGCTCCACGATC	310 bp	60°C
EVE-GT-internal-R	*Aedes aegypti* CFAV-EVE1 NS4B-NS4A region	detection and genotyping	reverse	CCGGTCACGCAGTTCTCCATTC	310 bp	60°C
EVE2-F	*Aedes aegypti* CFAV-EVE2 C region	detection	forward	CCTCATTCACATGGCATTGGTG	3417 bp	60°C
EVE2-R	*Aedes aegypti* CFAV-EVE2 NS2A region	detection	reverse	AGGAACTGTACGCACATCAGAG	3417 bp	60°C
EVE-SGup2-F	*Aedes aegypti* CFAV-EVE1 NS5 region close to upstream flanking region	sgRNA synthesis	forward	GAAATTAATACGACTCACTATAGGATGGGATCCCAGAAGCACGTTTTAGAGCTAGAAATAGC	124 bp	58°C
EVE-SGdwn1-F	*Aedes aegypti* CFAV-EVE1 NS2 region close to downstream flanking region	sgRNA synthesis	forward	GAAATTAATACGACTCACTATAGGAAGTATTCCTGACTTAAAAGGTTTTAGAGCTAGAAATAGC	124 bp	58°C
EVE-SGmid1-F	*Aedes aegypti* CFAV-EVE1 middle of NS2 region	sgRNA synthesis	forward	GAAATTAATACGACTCACTATAGGCGCTGGGGGAATCTGGGCGGGTTTTAGAGCTAGAAATAGC	124 bp	58°C
T7-R	-	sgRNA synthesis	forward	AAAAGCACCGACTCGGTGCCACTTTTTCAAGTTGATAACGGACTAGCCTTATTTTAACTTGCTATTTCTAGCTCTAAAAC	124 bp	58°C

Table S1. Primers used in this study. Related to Figure 1, 3 and 5.

Source				
published WGS data (SRR5562867)	CFAV-EVE1	CTCGCCATGGCACATCCCTGCTCCACGATCCTATAGCCGCATCATTTCGAACGTTGGCGATGAATTCTTCTTTTGATAAAATACGCGGTCGTAAGTTGCGATTCTTAAAGAACCGGATAAAATGCTTCATGATCGTCCTGTTGACTTTTCCAGAGCTCGAAACTGGTTGAAGGGGGCTACCGCGCCCAAGGCTCTAGCG<br>GCAATGTGCATGAACCTATAAAAAAGGTAGTTCATTTCTAGCAACCCTATGGCAGACACCATGGAGCCAAAATCCATGACATCATGGATCTTTGACCGGGAATGGAGAACTGCGTGACCGGTCTACAACTGAGCGCTCCCTTACCGTGCTAATGGCCCTGTCTCTCGCTACCGTTGTGTTTCTGATCTTCAGTTTGAAC<br>GCTATGTACAAGTACGCATGTCAATATCACAATCCGAGGAACGGCTACAGAGACTCTGGCGCGAATTTATGGTTTTGGACTGTATCCTTGGCGAGCGCTGGGGGAATCTGGGCGGCGGAGAAAGCTCACCAACCAACGGTGGCAGCTGTCCTAGCCTTTACAATGATAGTGCTCTTTCTTTACATGGAACAAACGAGC<br>GTTAGTATGGAGCTTGAGTTCATCTCAGCTGGAGAGACTCCCGAAGGAGTGTCAACCGAATATGATGACGGGATAAGTATTCCTGACTTA		
AaegL3 assembly	CFAV-EVE1 + flanking regions sequence	AACTACCAGAAACGGTTGTAATCGACAACGAAAAATCATTTTTATCCGGCGAACTTGTTAACTTTTTCAATGAAAACAAAATAACTCCTTATGTAGTAGCTACAGGTCGAAGCGAGATGAACGTGTGATATATGTCACAATCTGAATATATTATATACATGTAGTAGTGATAAGCAGAGAATAAAATAAACACAGCGAGCTG<br>GGCTCGCCCTCTTTTATCGTCGACTTTCGACCAAACAACACCTATTTTGTTGTACCTTCAATCCGAGAAACTAATAGCAGTAAACCTAAAAAGAACAATCAGTATAGAACATAAAAGTGGCGACGAGGATGGGATCCCAGAAGCACTGGTCCTGAATGGCTTCTTGCACATCTCGCCATGGCACATCCCTGCTCCACGAT<br>CCTATAGCCGCATCATTTCGAACGTTGGCGATGAATTCTTCTTTTGATAAAATACGCGGTCGTAAGTTGCGATTCTTAAAGAACCGGATAAAATGCTTCATGATCGTCCTGTTGACTTTTCCAGAGCTCGAAACTGGTTGAAGGGGGCTACCGCGCCCAAGGCTCTAGCGGCAATGTGCATGAACCTATAAAAAAGGTAG<br>TTCATTTCTAGCAACCCTATGGCAGACACCATGGAGCCAAAATCCATGACATCATGGATCTTTGACCGGGAATGGAGAACTGCGTGACCGGTCTACAACTGAGCGCTCCCTTACCGTGCTAATGGCCCTGTCTCTCGCTACCGTTGTGTTTCTGATCTTCAGTTTGAACGCTATGTACAAGTACGCATGTCAATATCACA<br>ATCCGAGGAACGGCTACAGAGACTCTGGCGCGAATTTATGGTTTTGGACTGTATCCTTGGCGAGCGCTGGGGGAATCTGGGCGGCGGAGAAAGCTCACCAACCAACGGTGGCAGCTGTCCTAGCCTTTACAATGATAGTGCTCTTTCTTTACATGGAACAAACGAGCGTTAGTATGGAGCTTGAGTTCATCTCAGCTG<br>GAGAGACTCCCGAAGGAGTGTCAACCGAATATGATGACGGGATAAGTATTCCTGACTTAAAAGTGGCGACGAGTTAAAACAGTGTTTAACGCGTGCAAAAGTTCATGTGAAAATCTAAGTGAATTTGTCTTTTGTCATCTTCCTGTGGAGTTTAATCACCGTTTAATTTCAATCTACTCGCAGCAGCATTTGGCCATTGCA		
Thai *Aedes aegypti* isofemale line	CFAV-EVE1 + flanking regions sequence	AACTACCAGAAACGGTTGTAATGGACAACGAAAAATCATTTTTATCCGGTGAACTTGTTAACTTTTTCAATGAAAACAAAATAACTCCTTATGTAGTAGCTACAGGTCGAAGCGAGATGAACTGTGATATATGTCACAATCAGATTATATTATATACATGTAGTAGTGATAAGCAGAGAATAAAATAAACACAGCGAGCTGG<br>GCTCGCTCTCTTTTATCGTCGACTATCGACCAAACAACACCTATCTTGTTGTACTTTCAATCCGAGAAACTAATAGCAGTAAACCTAAAAAGAACAATCAGTATAGAACATAAAAGTGGCGACGAGGATGGGATCCCAGAAGCACTGGTCCTGAATGGCTTCTTGCACATCTCGCCATGGCACATCCCTGCTCCACGATC<br>CTATAGCCGCATCATTTCGAACGTTGGCGATGAATTCTTCTTTTGATAAAATACGCGGTCGTAAGTTGCGATTCTTAAAGAACCGGATAAAATGCTTCATGATCGTCCTGTTGACTTTTCCAGAGCTCGAAACTGGTTGAAGGGGGCTACCGCGCCCAAGGCTCTAGCGGCAATGTGCATGAACCTATAAAAAAGGTAGT<br>TCATTTCTAGCAACCCTATGGCAGACACCATGGAGCCAAAATCCATGACATCATGGATCTTTGACCGGGAATGGAGAACTGCGTGACCGGTCTACAACTGAGCGCTCCCTTACCGTGCTAATGGCCCTGTCTCTCGCTACCGTTGTGTTTCTGATCTTCAGTTTGAACGCTATGTACAAGTACGCATGTCAATATCACAA<br>TCCGAGGAACGGCTACAGAGACTCTGGCGCGAATTTATGGTTTTGGACTGTATCCTTGGCGAGCGCTGGGGGAATCTGGGCGGCGGAGAAAGCTCACCAACCAACGGTGGCAGCTGTCCTAGCCTTTACAATGATAGTGCTCTTTCTTTACATGGAACAAACGAGCGTTAGTATGGAGCTTGAGTTCATCTCAGCTG<br>GAGAGACTCCCGAAGGAGTGTCAACCGAATATGATGACGGGATAAGTATTCCTGACTTAAAAGTGGCGACGAGTTAAAACAGTGTTTTACGCGTGCAAAAGTTCATGTGAAAATCTAAGTGAATTGGTCTTTTGTCATCTTCCTGTGGAGTTAAATCACCGTTTAATTTCAATCTACTCGCAGCAGCATTTGGCCATTGCA		
	CFAV-EVE1 + flanking regions sequence annotation	Positions on CFAV-EVE1 + flanking regions sequence	Positions on CFAV-KPP genome (LR596014)	Identity (%)
	Upstream flanking	1-329	-	-
	NS5	515-330	8563-8748	94.6
	NS4B	681-512	7322-7491	98.8
	NS4A	672-729	6324-6381	94.8
	NS2	726-1060	3924-4258	96.4
	Downstream flanking	1061-1200	-	-
published RNA-seq data (SAMN04480331, SAMN04480332, SAMN04480333)	CFAV-EVE2 sequence	GATAAATTTTTACTCTAAGCACCGCATCCAAATCGGGATGTCGGAATGCTTGTTTTAGTTCTTACAGGAGGACATCGTCCAAGCCCTCATTCACATGGCATTGGTGCTTCATGCCCTGTTTGCCTCCATCGACCGGCGAATTCGCTCATTATCTCGACGTGTTACCGCTCTTGAATCTCGACGAACCACAGGAAATCCTA<br>TGACGCTAGCTTTCATCTTGGGCTTTCTCACAGTGTTATGTGGATGCGTGGTGATTGACATGCAAGTTTCAACCGCTAAGGGTACGGAAATTTTTGATGGAAAGACGAACCGGACAGACTACTTGCATCTGTTCAAGTTACCCGCTGATGGTTGCTGGAGTGGCACTCTTGTCACAAAGAAGTGCCCAAAAGTGACTGA<br>CTTGGCAAAGGACCTCGAGTCGACAGATTGCGGATCAACGTGGACTGAGTTCACATTGCGGTATCGGAGATGCGTCGTGAAGAAACGTGAAAAGCGATCACGTGAACCCCCGAAAGCGGATCTGCTAGCTGAAATGGAGATCATCGCGTTCAAGACCATTCGCGAAAATAAGACAATTTTCATCGTGGCCCTATTGTG<br>TGTGGCAATCGCGAAGCGTTGGCCCACCTGGGTCGTGATTCTTCTGGCAATTGGCACTTGGACCACAGTAAAGGGGGAGTTTGTTGAACCGTTATACACGCTGAAAGCGGAACAGATGACCATGCTACAGACCATCGTGAGACCGGAGGAAGGGTACGTCGTGGCCACGCCAAACGGTCTTCTCGAGTTTAAAACA<br>GGACCGGCGGAGATCTATGGGGGGCAATGGCTGCGCGAGTTACTCGCAGATTGCCACGTCAACGCATCCTATTCGACTGACGTGTGTCCCGGTGGATCGCAGCTCAACATGGCTGACATCATGGCCAAGGAACGAGTATGCTCGACCCAACCATACAACCGCGGTTGGGGCACTGGGTGCTTCAAGTGGGGGATT<br>GGATTCGTTGGAACGTGCGTCGAACTCCATTGCGACAGAGGATTCAACGTTTCTTCTATCGCTAGGTCAGCCATTGTCATGAACGTTACGGCTAGCTTCCACTCCGTTAGCGACACACAACAGATGGTTGGCGACATACCGCTCACATTTAGGTTTGCGAAACTCGGAAACGCGGCGATGACCTGCAGGTTGGAGTCT<br>GAGCAGTTGCTGCTTGATTACTATCACGTTACGGGAAGCTCTCACGAGGGGCTGTTTTTACGGTCTCAAGTTGATAGTTGGCCTGGAGTGCACTCTACCGTTAGTGGTCGGCACGGCATGGAGAAAGTTGTTGTGTGGGGAGATGCTAGGTCCAATGAGATTCTAGTAAAAAACGTAATTGAACCTTCGTTGTCCTGG<br>GAAGACGCGATCGCCACCCATGATGGATTCCGTGACATCTCTTTTGTTTGTCAGATTATGCTCGACAAACTAGTCAGTGGTGCTTTTCGAGACTGTCCAGGCCCAAAAACATCCACATTTACCCAAGACGGCTTCGGGTATAGTGGAGTGGTAATAACTACCCTCACCGCTTCTTCTAACGAGACCTGTTCGCTTAGCTT<br>AACGTGCCACGGTTGTCTGCTGCAATCCACAAAAATGATCTTTCTCTCAGGGAAGACCACTTCTCGGGCATTTGTCAAGTGCGGGAACCATACAAGTACCTTGCTCGTTGGGAGCACTTCAGTTTCTATTGAGTGTGCCCTTAACCCTATCTCGCAAGGATGGCGCCTGGCTCGGCACGTGGTGGATCGGTATCGACG<br>GTTTGGCGTGTCGGGTGTCGCAGGAGTGTGGCAGGATTTGGTTGGAAAGTTTTCGGTTGGTGCCTTCTTCTCCAACACAACCCTTCTGGTCATTTTCGTCCTCGCTGCCCTGATTGACAAACGCATAGCCTTTCTATTTGTTTTAGGGGGGTATTTTTACTATGTGCGGGCTGATCTGGGGTGTGGAATTGACACCACG<br>AGAAAAACCATCTCGTGCGGCAGCGGCGCTTTTGTGTGGAAACACTTGGGCGTTGGCATTTCCAATGACCATGCTGTGGAACTTGAAGATTACTCGTTCACTGACTTGTACATAAAGGATATGTTCAGCTGGACCACGAAACCCTGCTTGATTTGTGAGGACGCTTTGCAGTGCGTAGCCCTGCGCAGAGCTGCTTTCT<br>CTGCTGTTGGGTCGATGGGTCCTGAACAGGTGTATGTAAATGACACATTGGCACGTACATTCAAGTTCTCCGAAACATCAAAGAGAACCATTTCCGTCACCATTAACCTAATTCAGTACAAGTTCTCATCTTATGTTGCACATGGACGCGCTGAGGGTGATTTGGGGCATCTACCAACAATGTTTGGAAGCTATCCCGAA<br>AAAGAGGCTGATAAGGTCATCCGGATTGTCGCATCGCGGCCCGATATTCGCCGACTTTGTGGAAAAGCTGTCTCCTTCCAATTCAAGTTTACGGGCTTCCGGCGAGGCCTGTATGGTTCAAACGTTCAGGTTGAGGTCTCAAAGAACTCATCGACCGAGTGTCCCACGTACCTGGCGGGCGTTGTCGTGAAGAATGG<br>TCGAACTGTCATCACTGACGGCATGTTTTGGATGGAAAGCACAGTGTTGGATGGAGTTGCTCAGATCACATCACTCGAGATGCGTCAATCACACCGCTGTGTGTGGCCCAGGGAATACACCCCGGACACGCTCAGTGACCCTTCAGACCAAGCTTTATTTATTCCACCTGCTTGGGGGGGACCAATTTCGCGAGTGAA<br>CCACATAATCGGGTACAAGACACAAACTGACTTTCCGTGGAACGTTAGTGACATCACTCTCATAGAGGGCCCAGCTCCTGGCACAAAGGTCAAGGTGGACTCTAGATGTCATGGGCGAATGCACGCACAGGTGATTGGACCGAATGACACGGAATCTTGGTGTTGCCAGTCATGCACAAGAATAGTCCACTTTCGAGT<br>CGGTGACCTCCTATATTATCCCATGGAGATCCAACTTGGAACCATGTCTGAAGCGTCAGAGTCGAATTCTAAAATATTTGAGGAACCGATCGGCGAGGAACCAGAACCAACAGTTGACGACATATTGAAGAGATACGGGAAGGCGAACGCGCAATCGGATTTCCGGAGGGTGAGCTCACGTGCAGGAGTTGGGTTCG<br>ATCGCTCCCTCCTAAATCTCCTGTGCCTCGCTATCTCATTGCAGCTTATCGGGGCAAAAACCAGAACATCGACACTAACTCGTCTATTTCTGACGATTCTAGCAATGGCGCTCTTTGGACTCCCCAATTTGTTTTCATCTGTGGGGCTATCTGCTTGGGTGTTGCTAGTGGCGTCCTCATCATCGCAACCGCAAGATCTA<br>TTGATGAATCTATGGATCATACTACAGACTGGGAGCTCAGCAGTCCTGCTGTTGGGGTACATGATCAGGAAGAAACTCTCAATAGTGCTAGGGGTCCACCACCTGGTGACTCTGATGTGCGTACAGTTCCTGTTTCCCGCCGTCGAGCCACC		
	CFAV-EVE2 sequence annotation	Positions on CFAV-EVE2 sequence	Positions on CFAV-KPP genome (LR596014)	Identity (%)
	C-NS2A	56-3516	331-3791	96.1

Table S2. CFAV-EVE1 and CFAV-EVE2 sequence annotation based on similarity to the CFAV-KPP genome. Related to Figure 1.

Repair template sequence	GAACAATCAGTATAGAACATAAAAGTGGCGACGAGGACGAGATCGCAGGAGGAAGTGATATCCGATGAGCATGGGAAGTGGCGACGAGTTAAAACAGTGTTTTACGCGTG		
Repair template annotation	Positions on CFAV-EVE1 + flanking regions sequence	Positions on repair template	
Upstream flanking HA	294-329	Jan-36	
CFAV-EVE1 NS5 modified	330-344	37-51	
Exogenous sequence	-	52-75	
CFAV-EVE1 NS5	1060	76	
Downstream flanking HA	1061-1094	77-110	
Guide sequences			
Name	Guide sequence	PAM	Strand
EVE-SGup2	GGATGGGATCCCAGAAGCAC	TGG	forward
EVE-SGdwn1	AAGTATTCCTGACTTAAAAG	TGG	forward
EVE-SGmid1	CGCTGGGGGAATCTGGGCGG	CGG	forward
Potential off-target sites			
EVE-SGup2	intergenic:AAEL008775-AAEL008778; intron:AAEL012020; intergenic:AAEL005724-AAEL005714; intergenic:Gap-AAEL014660		
EVE-SGdwn1	intergenic:AAEL008346-AAEL008344; intron:AAEL012182; intergenic:Gap-AAEL014360; intergenic:Gap-AAEL014457		
EVE-SGmid1	intron:AAEL003969; intergenic:AAEL003905-mir-999; exon:AAEL010467; intron:AAEL014570		
Deletion result: flanking regions without CFAV-EVE1 sequence	AACTACCAGAAACGGTTGTAATGGACAACGAAAAATCATTTTTATCCGGTGAACTTGTTAACTTTTTCAATGAAAACAAAATAACTCCTTATGTAGTAGCTACAGGTCGAAGCGAGATGAACTGTGATATATGTCACAATCAGATTATATTATATACATGTAGTAGTGATAAGCAGAGAATAAAATAAACACAGCGAGCTGGGCTCGCTCTCTTTTATCGTCGACTTTCGACCAAACAACACCTATTTTGTTGTACCTTCAATCCGAGAAACTAATAGCAGTAAACCTAAAAAGAACAATCAGTATAGAACATAAAAGTGGCGACGAGTTAAAACAGTGTTTAACGCGTGCAAAAGTTCATGTGAAAATCTAAGTGAATTTGTCTTTTGTCATCTTCCTGTGGAGTTTAATCACCGTTTAATTTCAATCTACTCGCAGCAGCATTTGGCCATTGCA		

Table S3. CRISPR/Cas9 design for CFAV-EVE1 knockout. Related to Figure 3.

	Head tissues						Ovary tissues					
	Day 4			Day 7			Day 4			Day 7		
	Df	***F***	***p***	***Df***	***F***	***p***	***Df***	***F***	***p***	***Df***	***F***	***p***
Experiment	4	5.47	0.0009***	2	1.17	0.3214	4	16.14	<0.0001***	2	9.88	0.0004***
Mosquito line	1	0.10	0.7585	1	10.43	0.0028**	1	4.53	0.0375*	1	40.09	<0.0001***
Experiment × Mosquito line	1	3.18	0.0204*	-	-	-	-	-	-	-	-	-

Table S4. Analysis of variance of CFAV RNA levels in tissues of CFAV-infected CFAV-EVE1 (+/+) and (-/-) *Aedes aegypti* mosquito lines. Related to Figure 5.

Library, SRA sample	Description	Total number of reads	Total number of reads mapped to CFAV	Number of reads mapped to CFAV, per 1 million reads
HGW27BGXX, SAMN13244317	Outbred Thai *Aedes aegypti* with CFAV-EVE1 (+/?) and CFAV-EVE2 (+/?), full body, naturally infected with CFAV	26244750	48779	1858.62
HG7CHBGXX, SAMN13244318	Outbred Thai *Aedes aegypti* with CFAV-EVE1 (+/?) and CFAV-EVE2 (+/?), full body, uninfected with CFAV	23582504	181	7.68
HVV5HBGXX, SAMN13244306	Thai Aedes aegypti isofemale line with CFAV-EVE1 (+/?), full body, CFAV IT injected, replicate 1	56026424	47262	847.57
HVV5HBGXX, SAMN13244307	Thai Aedes aegypti isofemale line with CFAV-EVE1 (+/?), full body, CFAV IT injected, replicate 2	52726002	50953	966.37
HVV5HBGXX, SAMN13244308	Thai Aedes aegypti isofemale line with CFAV-EVE1 (+/?), full body, CFAV IT injected, replicate 3	38976406	35066	899.67
HVV5HBGXX, SAMN13244303	Thai Aedes aegypti isofemale line with CFAV-EVE1 (+/?), full body, mock IT injected, replicate 1	68793501	450	6.54
HVV5HBGXX, SAMN13244304	Thai Aedes aegypti isofemale line with CFAV-EVE1 (+/?), full body, mock IT injected, replicate 2	33360151	339	10.16
HVV5HBGXX, SAMN13244305	Thai Aedes aegypti isofemale line with CFAV-EVE1 (+/?), full body, mock IT injected, replicate 3	95704497	504	5.27
HJ5CNBGX9, SAMN13244315	Isofemale line derived knockout line with CFAV-EVE1 (-/-), head, CFAV IT injected	18782124	15639	832.65
HJ5CNBGX9, SAMN13244313	Isofemale line derived knockout sister line with CFAV-EVE1 (+/+), head, CFAV IT injected	58304158	67711	1161.34
HJ5CNBGX9, SAMN13244316	Isofemale line derived knockout line with CFAV-EVE1 (-/-), ovary, CFAV IT injected	30427824	198	6.51
HJ5CNBGX9, SAMN13244314	Isofemale line derived knockout sister line with CFAV-EVE1 (+/+), ovary, CFAV IT injected	48147503	1327	27.56
HJ5CNBGX9, SAMN13244311	Isofemale line derived knockout line with CFAV-EVE1 (-/-), head, mock IT injected	46755526	32	0.68
HJ5CNBGX9, SAMN13244309	Isofemale line derived knockout sister line with CFAV-EVE1 (+/+), head, mock IT injected	20106002	150	7.46
HJ5CNBGX9, SAMN13244312	Isofemale line derived knockout line with CFAV-EVE1 (-/-), ovary, mock IT injected	45050253	10	0.22
HJ5CNBGX9, SAMN13244310	Isofemale line derived knockout sister line with CFAV-EVE1 (+/+), ovary, mock IT injected	28606142	522	18.25

Table S5. sRNA library information. Related to Figure 1, 2 and 4.
