# Supplementary figures and images for: Non-retroviral Endogenous Viral Element Limits Cognate Virus Replication in Aedes aegypti Ovaries
Source: Curr Biol. 2020 Sep 21;30(18):3495–3506.e6. doi: 10.1016/j.cub.2020.06.057 (PMC7522710; doi:10.1016/j.cub.2020.06.057)

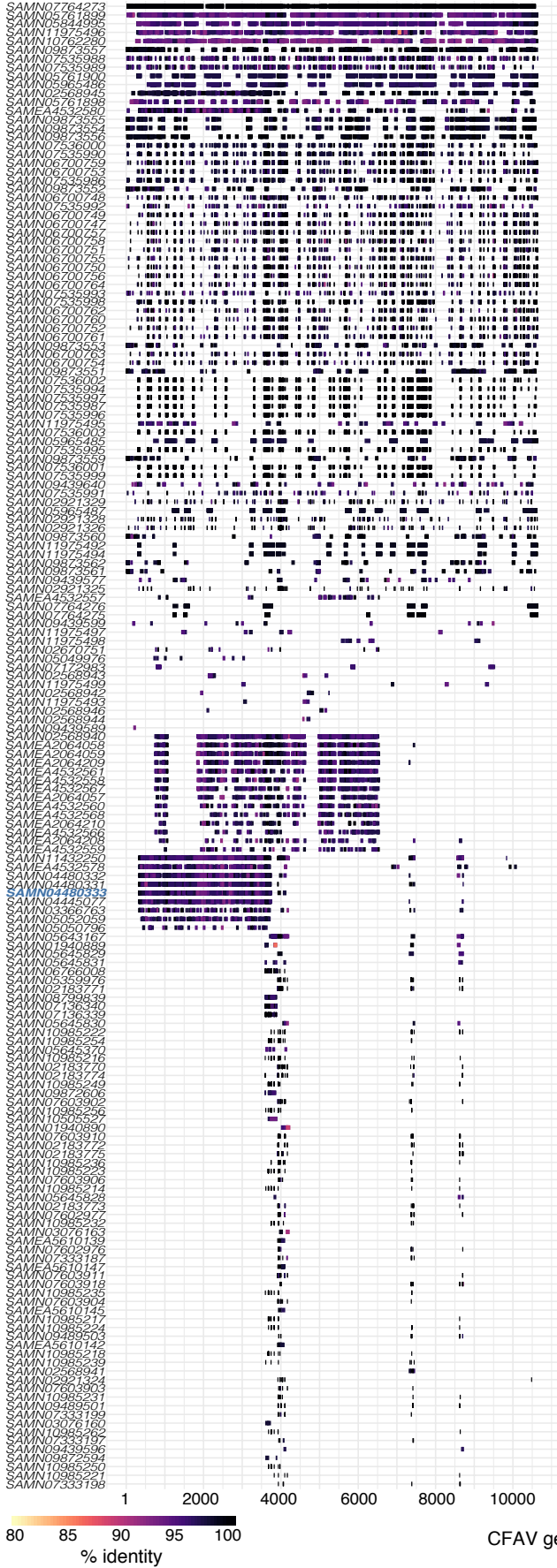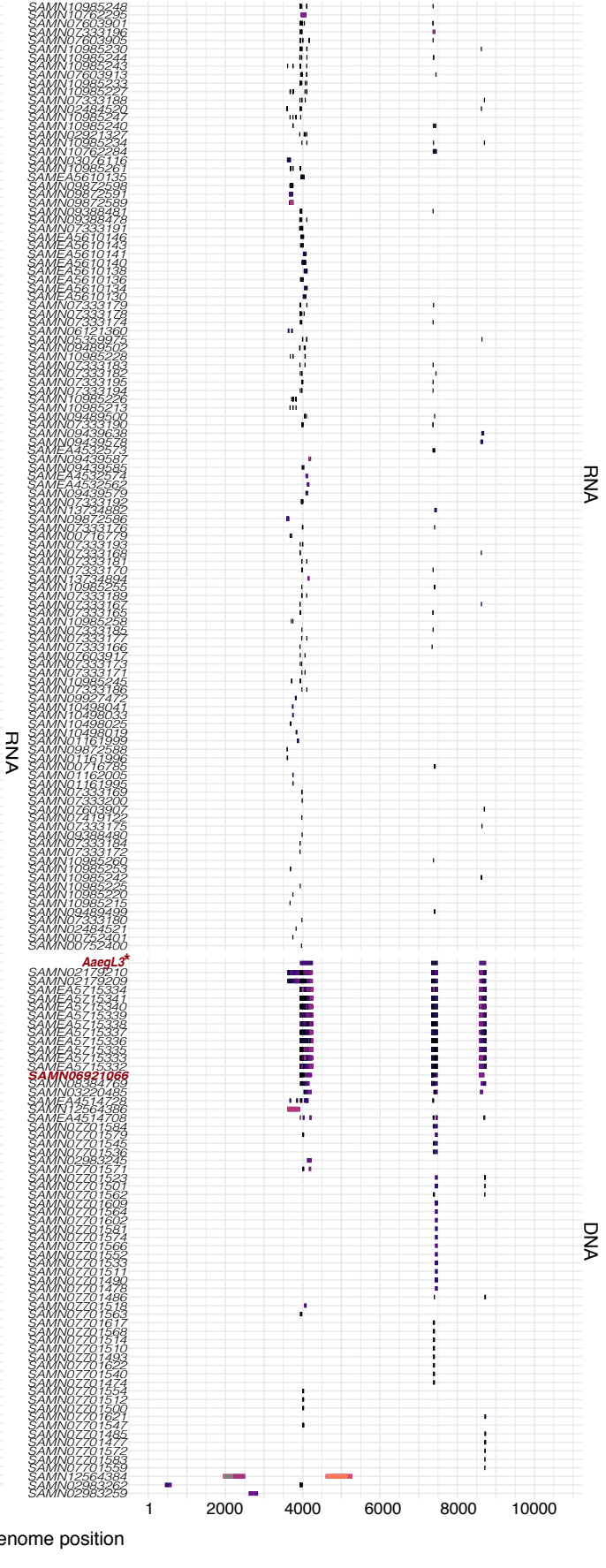

Supplement: Data S1. BLAST Alignment of Publicly Available Aedes aegypti Sequencing Data to the CFAV Genome, Related to Figure 1A — Each line represents a BLAST hit from a Sequence Read Archive (SRA) RNA-seq sample, an SRA whole-genome sequencing (WGS) sample, or the AeagL3 assembly (indicated by an asterisk). BLAST hits are aligned to the CFAV genome and ordered according to the pattern of coverage. The percentage of nucleotide identity to the CFAV-Bangkok (RNA) or CFAV-KPP (DNA) genomes is indicated by the color gradient shown at the bottom of the plot. The sample names colored in red and blue were used to reconstruct the sequence of CFAV-EVE1 and CFAV-EVE2, respectively. [file mmc2.pdf]
